# Functional carbon materials: effects and role of polymer-coating on carbon nanotubes

**DOI:** 10.1080/14686996.2025.2598944

**Published:** 2025-12-11

**Authors:** Naoki Tanaka, Masa-aki Morikawa, Tsuyohiko Fujigaya

**Affiliations:** a Department of Applied Chemistry, Graduate School of Engineering, Kyushu University, Fukuoka, Japan; b International Institute for Carbon Neutral Energy Research (WPI-I2CNER), Kyushu University, Fukuoka, Japan; c Center for Molecular Systems (CMS), Kyushu University, Fukuoka, Japan

**Keywords:** Carbon nanotubes, dispersant, non-covalent functionalization, polymer wrapping, catalyst, doping, sensor

## Abstract

Polymercoated carbon nanotubes (CNTs) provide defectfree interfacial control for sensors, thermoelectric, electrochemical and bio devices. We review roles of coated polymers for applications of polymer-coated CNTs for sensors, thermoelectric, batteries and biological applications.

## IMPACT STATEMENT

This review uniquely summarizes adsorption-driven polymer coating examples on carbon nanotubes (CNTs) and their roles in the various applications including sensors, thermoelectric conversion, batteries and biological applications.

## GRAPHICAL ABSTRACT

**Figure:**
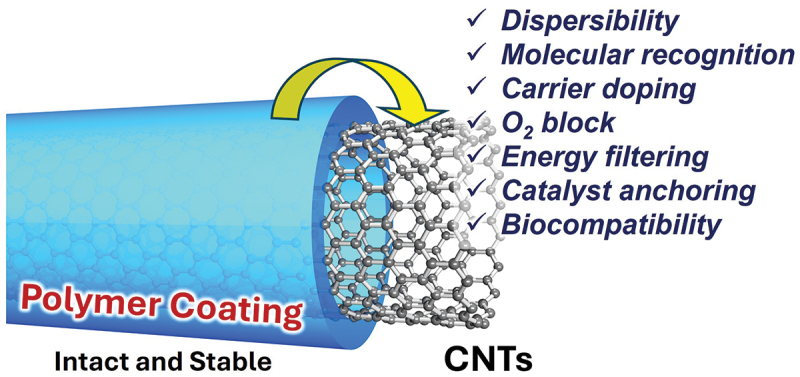

## 1. Introduction

### 1.1. Classification of carbon materials

**Figure 1.**
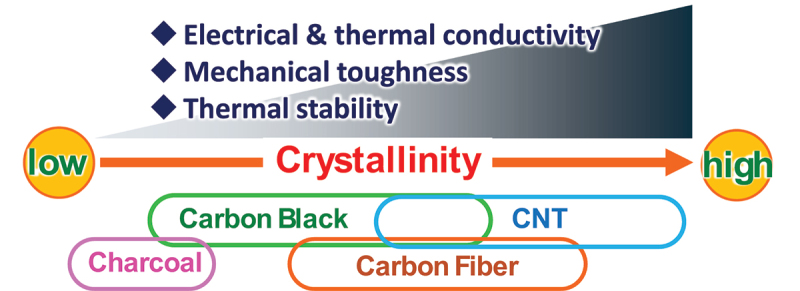
Types of carbon materials in relation to crystallinity and relationship between crystallinity and some representative carbon properties.

Carbon materials composed of sp^2^ -hybridized carbon atoms, such as graphite, activated carbon, and carbon Black (CB), have been utilized in a wide array of applications, including inks, tires, electrodes, and adsorbents [1,2]. These materials are often classified based on the extent of sp^3^ -type defects that they contain (i.e. the fraction of carbon atoms exhibiting sp^3^ hybridization) [3], which is commonly used as an inverse measure of their ‘crystallinity’. In this context, graphite and carbon nanotubes (CNTs) are considered highly crystalline because of their low sp^3^ -defect content, while materials like charcoal are regarded as low-crystallinity carbons as a result of the higher proportion of sp^3^ hybridization (Figure 1).

Carbon materials with low crystallinity have found widespread commercial use in commodity applications because of their low production costs and ease of processing [4]. In contrast, highly crystalline carbon materials have seen more limited adoption, primarily as a result of their high cost and strong tendency to form aggregates (bundles or stack), which is driven by van der Waals and π–π interactions. These interactions hinder their dispersion in solvents or matrices, which often calls for careful dispersion procedures. Nonetheless, the exceptional electrical and thermal conductivities, mechanical robustness, and thermal/electrochemical stabilities of highly crystalline carbons make them attractive candidates for improving the performance and longevity of advanced materials [5].

Among these highly crystalline carbon materials, CNTs have received considerable attention [6], not only because of their exceptional properties, but also because of their ability to form freestanding thin films with excellent flexibility and good mechanical strength [7], which can be used for device applications such as electrodes. Many of these unique natures of CNTs come from their one-dimensional (1D) nature of CNTs and are quite attractive than the other sp^2^-rich carbon materials such as graphene and fullerenes.

CNTs are typically classified into single-walled CNTs (SWCNTs) and multi-walled CNTs (MWCNTs), which have distinct structural and electronic properties that make them suitable for diverse applications in electronics [8], energy storage [9], biotechnology [10], and advanced composites [11,12]. In particular, the one-dimensional structure of SWCNTs offers unique light absorption/emission characteristics in the near-infrared (NIR) region, which have attracted considerable attention for bioapplications because NIR light does not overlap with the absorption/emission of tissues [13].

### 1.2. Surface modification of CNTs

**Figure 2.**
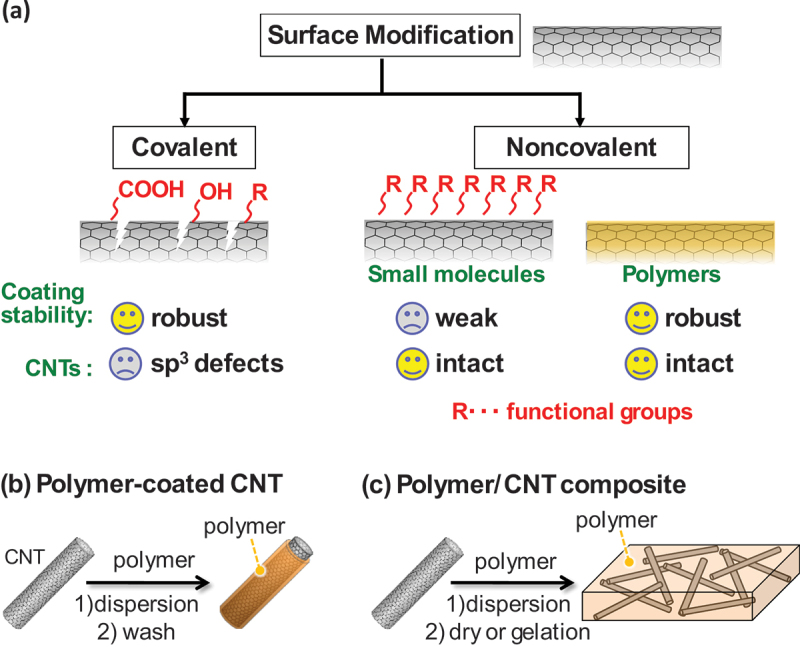
(a) Schematic illustration of surface modification classification and their characteristics for covalent and noncovalent approach. These represent the stable coating of polymer and intactness of CNTs for polymer coating approach. (b, c) Examples of the preparation of polymer-coated CNTs (b) and polymer/CNT composites (c). Polymer-coated CNT only consists of the polymer adsorbed on CNT surface while polymer/CNT composite contains polymer not adsorbed on CNT surface.

Effective dispersion strategies have been extensively studied to fully exploit the advantages of CNTs in practical applications. The dispersion of CNTs typically begins with a mechanical dispersion technique such as ultrasonication, high-shear mixing, high-pressure homogenization, or ball milling, which disrupts the CNT aggregates by applying mechanical energy. Either covalent or noncovalent surface modification is then commonly employed to prevent the reaggregation of these dispersed CNTs (Figure 2(a)) [14–16].

Covalent functionalization typically begins with oxidative acid treatment (e.g. using a HNO_3_/H_2_SO_4_ mixture) to introduce functional groups such as carboxylic acids or hydroxyl groups on the CNT surfaces [17]. These groups are often used as anchor points for further chemical reactions such as amidation and esterification. In addition, π-chemistry, which was primarily developed for fullerene modification, has also been used for CNT surface modification [18]. While covalent methods offer durable and robust functionalization, they inevitably introduce sp^3^ defects into the carbon lattice, potentially degrading intrinsic CNT properties [19] and thus necessitating careful optimization.

In contrast, noncovalent functionalization relies on physical interactions such as π–π stacking, hydrogen bonding, and hydrophobic effects to immobilize molecules on the CNT surfaces without destroying their sp^2^ carbon skeleton [20–22]. In this approach, surfactants, small molecules, and polymers are employed to form physically adsorbed coatings, which increase the wettability and dispersion stability of the CNTs in solvents or polymer matrices [23,24]. These methods are relatively straightforward and typically involve mixing CNTs with dispersant molecules under strong shear forces (e.g. using ultrasonication, ball milling, or jet milling).

In surfactant-assisted dispersion, an anionic surfactant such as sodium dodecyl sulfate (SDS), a bile salt derivative such as sodium deoxycholate (DOC), or a polysaccharide such as carboxymethyl cellulose (CMC) is commonly used to disperse the CNTs in an aqueous medium by imparting a surface charge and/or steric stabilization [25]. In addition to the fundamental research on CNTs and their applications, such dispersions are often employed as starting points for subsequent covalent functionalization to ensure homogeneous reaction conditions. However, it is important to note that the surfactants adsorbed on the CNT surface exist in dynamic equilibrium with free surfactants in the surrounding solution [26]. Consequently, the removal of free surfactants by filtration or dialysis often leads to the desorption of surfactants from the CNT surface and subsequent reaggregation of CNTs [27].

In contrast, the polymers used in noncovalent functionalization often exhibit multiple interaction sites on the CNT surface, resulting in more irreversible adsorption (Figure 2(b)). In such cases, the adsorbed polymers remain attached to the CNTs even after extensive washing, forming polymer-coated CNTs with coaxial core – shell structures that exhibit significantly enhanced dispersion stability. While the presence of polymer coatings may be considered as unwanted contamination in some contexts, carefully designed polymers can preserve and even enhance CNT performance by imparting additional functionality. Because of their structural tunability and versatility, polymer-coated CNTs have recently attracted increasing interest and are being increasingly adopted in the design of CNT-based materials.

Various polymers have been reported for use as CNT dispersants, including DNA [28], π-conjugated polymers [29–31] such as poly(3-hexylthiophene) (P3HT) and poly(9,9-dioctylfluorene) (PFO), and aromatic polycondensates such as polybenzimidazole (PBI) and polyimide [18,22,32–42].

In another type of CNT material hybridized with a polymer, CNTs are embedded in the polymer matrix (Figure 2(c)) [43]. Polymer-coated CNTs are typically prepared by dispersing CNTs in a polymer solution, filtering the dispersion, and washing the filtered material with a polymer solvent. CNT/polymer composites have also been prepared by drying or gelation after casting. These composite materials are here categorized as CNT/polymer composites and are not the main focus of this review.

### 1.3. Mechanism of adsorption-based polymer coating

Despite the growing interest in and diverse applications of polymer-coated CNTs, systematic studies on their adsorption behaviors, such as their adsorption kinetics, thermodynamics, coverage ratios, and coating thicknesses, remain limited, as is the case with polymer coatings on other materials. Adsorption phenomena of polymers on carbon can be extrapolated based on the studies of polymer adsorption on solid surface. Polymer adsorption onto solid surface is characterized by their adsorption at very low concentrations compared to that of low molecular weight molecules, therefore the concentration of polymers for CNT dispersion can be much lower compared to that of low molecular compounds. And adsorption of polymers on solid materials is generally irreversible because of multipoint interactions but can be replaceable when higher molecular weight polymers or other adsorbates exist. In addition, in many cases, polymer adsorption on solid materials was analyzed as entropy-driven process, where entropy-gain arouses from the detachment of the solvent molecules from the surface and polymer chains dominate over the entropy-penalty originated from the loss of chain flexibility upon the adsorption. But attractive forces such as electrostatic, π-π stacking and van der Waals interaction can also contribute to the progress of the adsorption. Therefore, interactions at solvent molecules/solid surface interface and solvent molecules/polymer interface play key role, suggesting choice of solvents is crucial and solubility parameters such as Hansen parameters are quite useful to systematically understand the adsorption events. We also need to recognize that adsorption of polymer onto solid materials does not always mean the dispersion of polymer-coated solid materials; namely aggregation of the polymer-coated solid materials takes place when the polymer-polymer interaction works predominantly rather than polymer-solvents interaction.

To date, adsorption studies using CB as the adsorbent have been experimentally conducted to address the influence of the polymer composition [44] and molecular weight [45]; the thickness of the adsorbed polymer layer has also been studied [46,47]. We investigated the adsorption of PBI onto CB using adsorption isotherm measurements at different temperatures. The adsorption process was irreversible and promoted at higher temperatures, indicating that PBI adsorption was entropy-driven [48]. Atomic force microscopy (AFM) measurements determined that the thickness of a PBI coating on highly ordered pyrolytic graphite (HOPG) was approximately 0.45 nm, and similar coating morphologies were found on both CB and CNTs. Transmission electron microscopy (TEM) analysis of the PBI-coated CNTs revealed a coating thickness of approximately 0.5 nm [49]. Assuming a uniform coating thickness of approximately 0.45 nm, the surface coverage ratio of PBI at saturation was estimated to be approximately 50–60% [48]. For the CNTs, we also used SEM to observe the partial surface coverage of PBI [50]. Although the exact mechanism for this incomplete coverage remains unclear, we hypothesize that full surface coverage is inherently difficult because of the random nature of polymer adsorption on carbon surfaces.

### 1.4. Polymer coating via electrochemical polymerization

Another promising approach to fabricate polymer-coated CNTs is coating of CNTs based on electrochemical polymerization of monomers such as pyrrole [51,52], aniline [53] and 3,4-ethylenedioxythiophene (EDOT). In polymer coatings based on electrochemical polymerization, the coating coverage and thickness can be tuned by varying the polymerization time, whereas adsorption-based techniques only allow monolayer coatings. Although this approach is limited to monomers that undergo oxidative polymerization, it differs from adsorption-based coatings in that specific monomer –CNT interactions are unnecessary: the redox reaction is confined to the CNT surface, so the polymer forms selectively on the nanotube. A further advantage is substrate compatibility – freestanding CNT sheets or fibers can serve as the working electrode for the polymerization, and the resulting coated sheets or fibers can be used directly in applications. These approaches are summarized in the other review article and out of main focus of this review [54].

## 2. Applications for polymer-coated CNTs

### 2.1. Sensors

SWCNTs possess large specific surface areas and excellent carrier mobility and are highly sensitive to even slight changes in the surrounding environment, making them highly effective sensing materials [55]. In recent years, progress in understanding the sensing mechanisms of SWCNTs and advancements in electrical interfaces have driven the development of a wide range of sensors, including gas sensors [56–61], ion sensors [62–65], and biosensors [66–74]. Nevertheless, achieving SWCNT-based sensors with high sensitivity and selectivity toward specific target analytes remains a critical issue in sensor research.

The sensing performance of SWCNTs can be enhanced by physical or chemical functionalization [55]. Among these methods, polymer coating enables a high response sensitivity because of its ability to achieve high dispersion without inducing significant defects in the SWCNT structure. More importantly, the intrinsic adsorption and coordination sites of polymers can serve as active sites for the direct capture of target analytes or introduction of functional groups to facilitate their recognition. In such applications, polymer-coated CNTs have a preferable morphology compared to polymer/CNT composites, in which polymer-coated CNTs possess diffusion paths for analytes inside the CNT network.

This section discusses the role of the polymer layer in gas and ion sensors as representative small-molecule sensing platforms. The focus is primarily on sensors that operate via electrical response detection [75,76] and field-effect transistor (FET) sensors [77,78].

#### 2.1.1. Gas sensor

Dai et al. reported a pioneering work on chemoresistive sensors based on non-coated SWCNTs [79]. In recent years, there has been a surge in the demand for gas sensors with higher sensitivity and selectivity owing to growing environmental awareness, expanding industrial needs, and heightened concerns for health and safety [80]. From this perspective, SWCNT-based gas sensors have advantages compared to conventional metal-oxide semiconductors [81] because they operate at room temperature. However, improving the sensitivity and selectivity of gas detection remains a challenge.

The use of high-purity semiconducting SWCNTs (sc-SWCNTs) is a promising approach for enhancing gas-sensing performance [82–84]. Recent advances in separation techniques have made it possible to extract sc-SWCNTs from mixed SWCNT samples, thus promoting their practical application at a lower cost [85–88]. Among the various separation techniques, the selective polymer coating of sc-SWCNTs using conjugated polymers is considered one of the most promising methods for scalable and selective extraction [87,89]. Recently, polymers containing imine bonds (–CH = N–) have been widely used for the selective extraction of sc-SWCNTs. The resulting polymer-coated SWCNTs have been employed as chemiresistive sensors for NH_3_ and NO_2_, demonstrating detection limits in the parts-per-billion range [90–94].

From the perspective of polymer design, an improved gas-detection performance can be achieved by employing polymers that possess binding sites capable of selectively interacting with the target gas. For example, polyethylenimine (PEI) is effective for CO_2_ capture and storage [95–99]. Leveraging this advantage, CO_2_ sensors constructed from PEI-coated SWCNTs have been reported [100,101]. In contrast, nonpolar gases such as hydrogen and methane exhibit weak interactions with polymers and SWCNTs. One emerging strategy involves the immobilization of a catalyst on the surface of SWCNTs to enable the detection of analyte chemical reactions. Swager et al. utilized poly(4-vinylpyridine) (P4VP)-coated SWCNTs, in which the pyridyl group of P4VP served as an anchoring site to immobilize platinum via Lewis basicity, thereby successfully achieving methane detection [102,103]. They also reported that iptycene-containing poly(arylene ether)s limited the growth of palladium nanoparticles (PdNPs) and stabilized their dispersion, offering sensor materials with high sensitivity, selectivity, and robustness toward hydrogen gas [104]. Table 1 summarizes the gas sensors based on polymer-coated SWCNTs reported since 2020 [90–94,100–108]. Detailed discussions of the response mechanisms of SWCNT-based gas sensors are provided in another article [60].

**Table 1.** Polymer/SWCNT-based sensors reported after 2020.

Polymer/ SWCNT	Chemical structure of polymers	Detected gases	Sensor type	Limit of detection	Ref.
FO-N-PA/ SWCNT	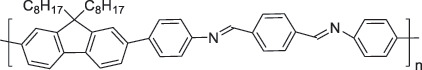 FO-N-PA	NH_3_	Chemiresistive	75.78 ppb	[90]
PEI-PEG/ SWCNT	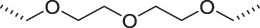 PEG 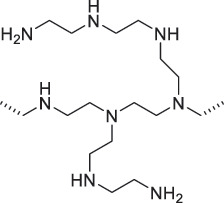 PEI	CO_2_	Chemiresistive	N.A.	[100]
Pent-SO_2_-Pd/ P2-SWCNT	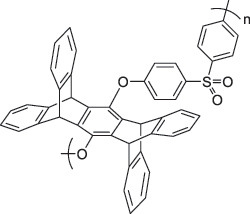 Pent-SO_2_ 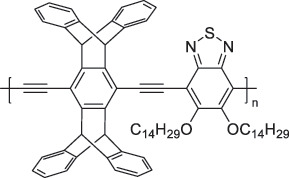 P2	H_2_	Chemiresistive	91 ppm	[104]
PF2L/ sc-SWCNT	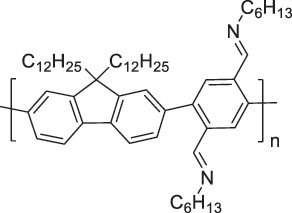 PF2L	NH_3_ NO_2_	Chemiresistive	4.265 ppb (NH_3_) 1.471 ppb(NO_2_)	[91]
PDF-DTS/ SWCNT PDF-N-DTS/ SWCNT	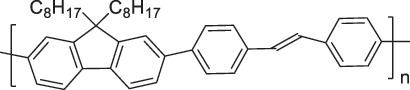 PDF-DTS 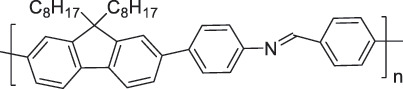 PDF-N-DTS	NH_3_	Chemiresistive	64.5 ppb (PDF-DTS) 53.5 ppb (PDF-N-DTS)	[92]
PFDD/ SWCNT	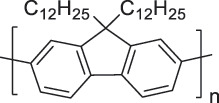 PFDD	NO	FET	N.A.	[105]
PFID/ SWCNT	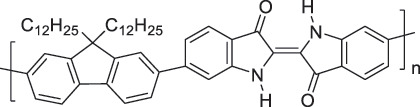 PFID	CO_2_	Chemiresistive	N.A.	[106]
PBC-DTS/ SWCNT PBC-N-DTS/ SWCNT	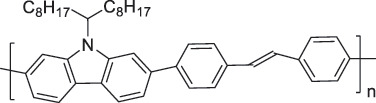 PBC-DTS 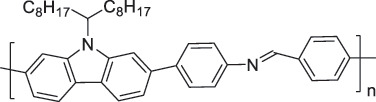 PBC-N-DTS	NH_3_ NO_2_	Chemiresistive	NO_2_1.34 ppb(PBC-DTS) 4.41 ppb (PBC-N-DTS) NO_2_ 22.7 ppb (PBC-DTS) 41.3 ppb (PBC-N-DTS)	[93]
PEI-Starch/ SWCNT	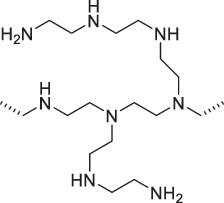 PEI 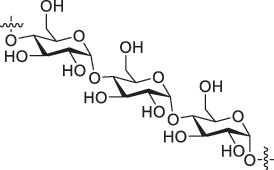 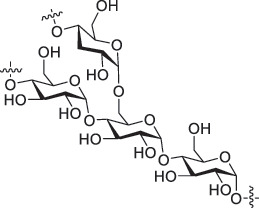 Starch	CO_2_	Chemiresistive	N.A.	[101]
PMMA- P(FD-N3)-Pd/ SWCNT	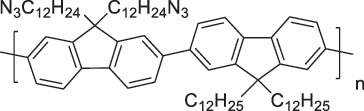 P(FD-N3)	H_2_	Chemiresistive	N.A.	[107]
Polymeric ion liquid/ SWCNT	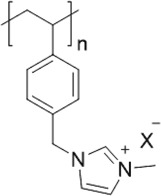 X^–^ = Cl^–^, BF_4_^–^, PF_6_^–^, Tf_2_N^–^	SO_2_	Chemiresistive	20.4 ppt	[108]
PFOB/SWCNT PFDB/SWCNT PFTB/SWCNT	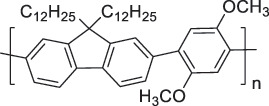 PFOB 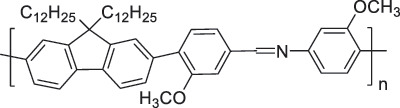 PFDB 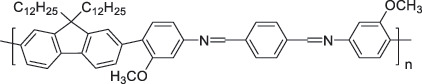 PFTB	NH_3_	Chemiresistive	0.0126 ppm (PFOB) N.A. (PFDB) 0.0130 ppm (PFTB)	[94]
P4VP-Pt-POM/SWCNT	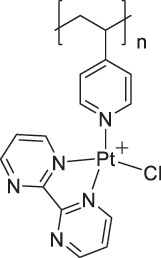 P4VP-Pt-POM [H_5_PV_2_Mo_10_O_40_]^–^ (POM)	CH_4_	Chemiresistive	29 ppm	[102]
PyBr-R_1_/ SWCNT	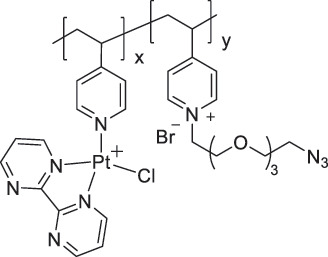 PyBr-R_1_ [H_5_PV_2_Mo_10_O_40_]^–^	CH_4_	Chemiresistive	N.A.	[103]

#### 2.1.2. Ion sensor

Ion sensors are utilized for the detection of specific ions in various fields owing to their high sensitivity and selectivity. In the medical field, for instance, they are used to measure electrolytes in blood and urine, as well as to monitor intracellular ions, thereby contributing to diagnostics and the analysis of physiological functions [109]. In the fields of food and agriculture, ion sensors play a vital role in areas closely tied to daily life, such as crop-nutrient management and food-product quality assessment [110].

**Figure 3.**
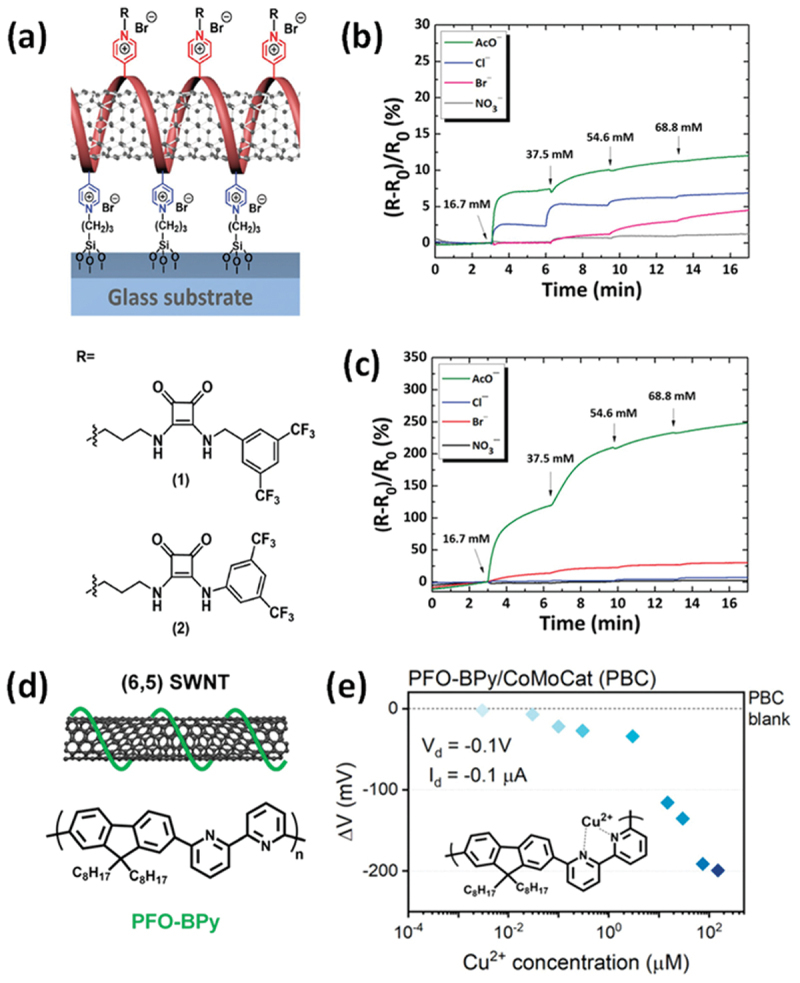
(a) Schematic illustration of the chemical structure of immobilized P4VP-SWCNTs with functionalized anion-binding selectors. Sensitivities of (b) P4VP-1-SWCNTs and (c) P4VP-2-SWCNTs toward anions in a concentration range of 16.7 × 10^−3^–68.8 × 10^−3^ M in acetonitrile (CH_3_CN). Reproduced by permission from [62], copyright 2019, wiley-VCH GmbH. (d) Molecular structure of PFO-BPy/SWCNTs. (e) Correlation of voltage shift (δv) with increasing Cu_2_^+^ concentration at a fixed drain current of −0.1 μA, with inset showing possible interaction of Cu^2+^ with bipyridine unit of PFO-BPy. Reproduced by permission from [120], copyright 2025, royal society of chemistry.

Ion detection requires the use of various types of intermolecular interactions, such as coordination with a metal center, ion pairing, and hydrogen bonding. Swager et al. developed anion sensors based on P4VP-coated SWCNTs [62,65]. When anion selectors are coordinated with the pyridine moieties of P4VP, the formation of pyridinium groups enhances the anion-binding affinity of the selectors (Figure 3(a)) [62]. P4VP-SWCNTs functionalized with selector 1 (P4VP-1-SWCNT) exhibited higher sensitivity for AcO^−^ than for Cl^−^ > Br^−^ > NO_3_^−^. However, better sensitivity was obtained with P4VP-2-SWCNT, which was attributed to the internal charge transfer transition resulting from the deprotonation of the selector (Figures 3(b,c)). Chemiresistive sensor arrays have been integrated with a wireless sensing module and demonstrated the real-time detection of multiple anions in small-volume samples (2 µL).

Similarly, anion sensing was achieved using thiourea-based dual hydrogen bond donors as selectors coordinated with pyridinium moieties [65].

Highly dispersed, high-purity sc-SWCNT inks enable the fabrication of electronic devices and FETs using methods such as inkjet printing [111,112], aerosol jet printing [113–115], spin coating [116], and dip coating [117]. Therefore, poly[(9,9-dioctylfluorenyl-2,7-diyl)-alt-(6,6’-[2,2’-bipyridine])] (PFO-BPy), which is a conjugated polymer that selectively wraps sc-SWCNTs, has been used as a high-purity SWCNT ink (Figure 3(d)) [118,119]. Zaumseil et al. exploited the coordination capability of pyridine moieties in PFO-BPy/SWCNTs to fabricate a water-gated transistor capable of selectively detecting Cu^2+^ ions [120]. The presence of positively charged Cu^2+^ ions near the SWCNTs inhibited hole accumulation, resulting in a shift in the transfer curve toward more negative gate voltages. This shift enabled the quantitative detection of Cu^2+^ concentrations ranging from 0.1 µM to 100 µM (Figure 3(e)). They also revealed that replacing the alkyl groups on the fluorene moieties of PFO-BPy with tetraethylene glycol side chains enhanced the device performance [121].

**Figure 4.**
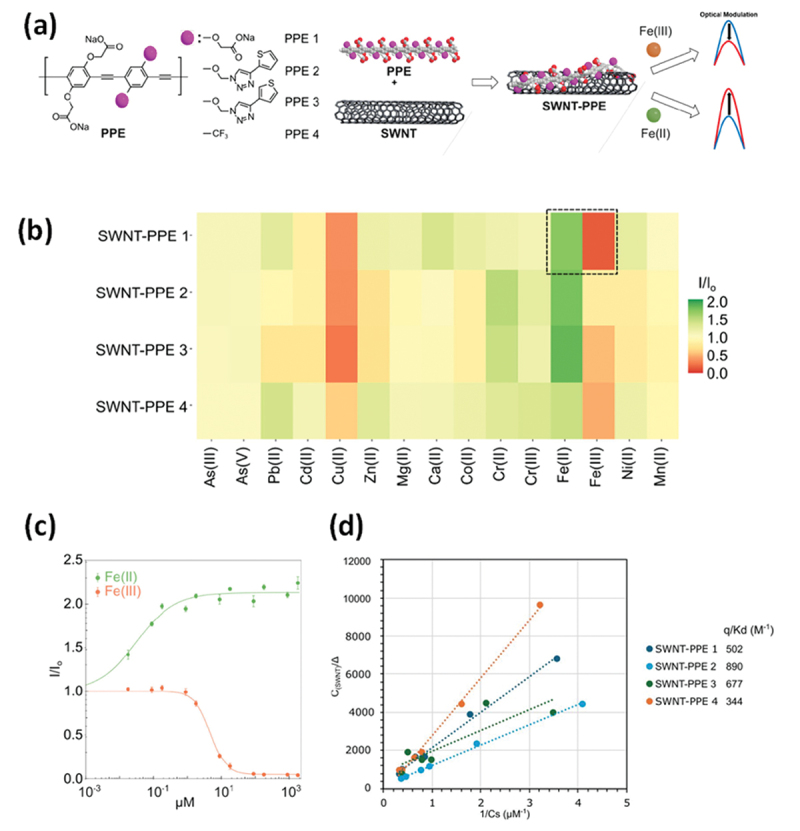
Development of SWCNT-PPE as ion nanosensor. (a) Schematic diagram showing the structure of the SWCNT/PPE complex and its function as an ion nanosensor. (b) False-color heatmap summarizing the optical modulation of SWCNT/PPE 1–4 to a library of metal ions. (c) Calibration curves of SWCNT/PPE 1 to Fe(II) and Fe(III). Error bars indicate the standard deviation from three independent measurements. (d) MPA analysis results, with linear fitting of SWCNT/PPE (1–4) corona phase data performed based on an adsorption site balance model. The q/Kd (M^−1^) parameter provided insights into the surface coverage of the corona on the nanotubes. A higher q/Kd value generally implies a looser, less compact corona phase with greater accessibility for probe binding, while a lower q/Kd indicates a tighter, more densely packed corona phase. Reproduced by permission from [124], copyright 2025, American Chemical Society.

Strano et al. developed a technique for corona-phase molecular recognition (CoPhMoRe) that utilizes the NIR response of SWCNTs functionalized with synthetic polymers [122,123]. An NIR fluorescent nanosensor was developed using SWCNTs coated with an anionic poly(*p*-phenylene ethynylene) (PPE) polyelectrolyte, enabling the distinction between Fe (II) and Fe(III) species within plant tissues at a low detection limit of 10 nM (Figure 4(a)) [124]. The corona phase formed by the PPE1-coated SWCNTs showed an ‘on’ NIR fluorescence response toward Fe (II) and ‘off’ response toward Fe(III), while exhibiting minimal reactivity toward other metal ion species (Figure 4(b)). Although the exact mechanism remains to be elucidated, it is evident that nonspecific fluorescence quenching occurs between PPE and various metal ions. Furthermore, the structural modifications of the PPE polymer did not lead to significant changes in the optical modulation profiles (Figures 4(a,c)).

To investigate the strength of the interaction between the SWCNTs and PPEs, the surface coverage parameter (q/Kd) was measured using the molecular probe adsorption (MPA) technique [125]. The q/Kd values for the four types of PPE-coated SWCNTs varied between 344 and 890 M^−1^. These results suggest that the ion-selective response arises not from the structural composition of the polymer but from the intrinsic properties of the corona phase interactions.

### 2.2. Thermoelectric materials

Thermoelectric generators (TEGs) convert temperature gradients into electricity and have attracted significant attention as energy-harvesting devices because of their simple structure, which does not involve moving parts. A typical TEG module consists of a π-shaped assembly of alternating p-type and n-type thermoelectric (TE) materials, electrically connected in series and thermally in parallel. Inorganic materials such as bismuth telluride (BiTe) have been widely used. Recently, CNTs have emerged as promising candidates for TE materials, particularly for wearable TEGs, because they are lightweight and structurally flexible, even when formed into relatively thick network sheets. Consequently, CNT-based TEG modules can conform to heat-source surfaces and effectively utilize ubiquitous waste heat, such as body heat [126,127].

In the 1990s, the possibility of using nanostructuring to control various physical properties, including the Seebeck effect, was suggested. A large Seebeck effect has been predicted for low-dimensional materials [128]. In this context, SWCNTs, particularly sc-SWCNTs, are regarded as promising TE materials owing to their excellent electrical and mechanical properties. Numerous studies have been conducted on SWCNT-based TE materials [127,129–132].

Polymer-coated SWCNTs have primarily been used for SWCNT-based TE applications because they allow solution-based fabrication and enable the incorporation of polymer properties to enhance the TE performance. Various polymers have been used as coating agents, including vinyl and semiconducting polymers such as polythiophenes [133–147]. This section highlights the role of polymer coatings in enhancing the thermoelectric performance of CNTs, with particular focus on SWCNT networks, where the SWCNTs form a continuous sheet structure.

#### 2.2.1. Doping

Although SWCNTs are inherently n-type, environmental oxygen readily p-dopes them owing to their band structure, making them p-type [148]. Therefore, doping techniques that switch the carrier type from holes (p-type) to electrons (n-type) are necessary. Owing to the advancements in organic semiconductor doping, n-type SWCNTs can now be readily prepared by injecting electrons into p-type SWCNTs. Organic and inorganic small molecules have traditionally served as electron injectors (n-dopants), and polymers have been used since the early 2000s because of their chemical stability.

**Figure 5.**
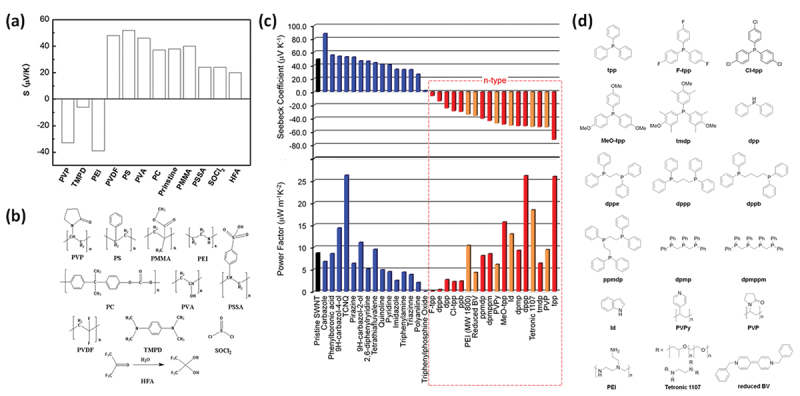
(a) Seebeck coefficients of SWCNT film doped with the various molecules shown in (b). A positive value for the Seebeck coefficient indicates a p-type material, while a negative value indicates an n-type material. (b) Chemical structures of the dopants shown in (a). Reproduced by permission from [150], copyright 2012, wiley-VCH GmbH. (c) Seebeck coefficients (upper panels) and power factors (lower panels) of SWCNT films doped with the various molecules shown in (d). (d) Chemical structures of the dopants shown in (c). Reproduced by permission from [151], copyright 2013, Springer nature limited.

In 2011, Yu et al. demonstrated the electron doping of SWCNTs using polyethyleneimine (PEI), achieving a PEI-coated SWCNT film with a negative Seebeck coefficient of −58 µV/K [149]. Dettlaff -Weglikowska et al. prepared various polymer-coated SWCNT films by immersing them in polymer solutions such as poly (vinylpyrrolidone) (PVP) (Figures 5(a,b)) [150]. They found that poly(styrene sulfonic acid) (PSSA) facilitated p-doping, likely as a result of proton donation from sulfonic acid groups, whereas PVP promoted n-doping via electron donation. Nonoguchi et al. systematically studied dopants, ranging from small molecules to polymers [151]. The undoped Seebeck coefficient of +49 μV/K at 310 K changed dramatically after doping, ranging from +90 μV/K to −80 μV/K. In addition to amine-based dopants, phosphine derivatives provide relatively stable n-type doping (Figure 5(c,d)). A clear correlation was observed between the work function of the film and highest occupied molecular orbital (HOMO) energy level of the dopant, as measured by atmospheric photoelectron yield spectroscopy. These results indicated that intermolecular charge transfer upon adsorption was the primary doping mechanism.

**Figure 6.**
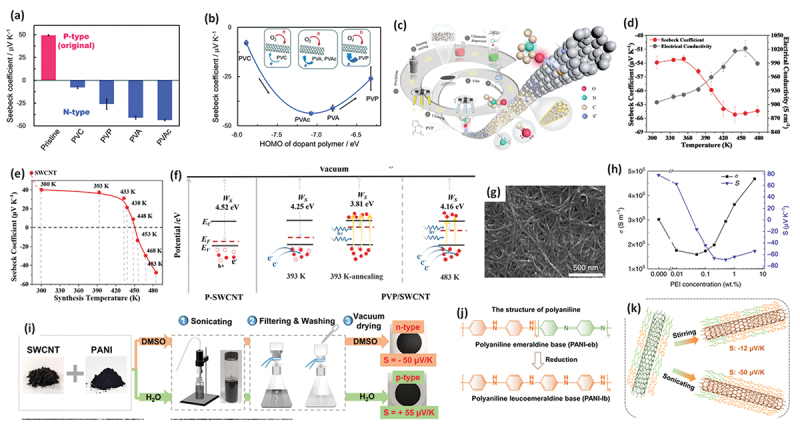
(a, b) Seebeck coefficients of SWCNT thin films doped with PVC, PVP, PVA, and PVAc at 300 K (a) and their Seebeck coefficient – HOMO level plot (b). Reproduced by permission from [152], copyright 2025, scimago journal & country rank. (c) Schematic illustration of PVP-coating of SWCNTs. (d) Temperature dependence of Seebeck coefficient (red plots) and electrical conductivity (Black plots) for PVP-coated sWcnts. (e) Room-temperature Seebeck coefficients of the PBI-coated SWCNT films synthesized at different temperatures. (f) Band diagrams of the PVP-coated SWCNTs illustrating the mechanism of the temperature-dependent p-n conversion. Reproduced by permission from [153], copyright 2024, wiley-VCH GmbH. (g) SEM image of the SWCNT sheet after the PEI doping. (h) Concentration dependence of Seebeck coefficient (blue plots) and electrical conductivity (Black plots) for PVP-coated SWCNTs. Reproduced by permission from [154], copyright 2017, Springer nature limited. (i) Illustration of the PANI-coated SWCNT hybrid film preparation process. (j) The molecular structure of polyaniline. (k) Schematic illustration of PANI-coating depending on the treatment condition. Reproduced by permission from [155], copyright 2025, royal society of chemistry.

Horike et al. further explored the HOMO-level correlation using vinyl polymers such as poly (vinyl alcohol)(PVA), poly(vinyl acetate) (PVAc), and poly(vinyl chloride) (PVC) (Figure 6(a)) [152]. They demonstrated that higher HOMO levels in the side-chain structures correlated with more effective electron injection and increased the absolute value of the negative Seebeck coefficient (from PVC to PVAc), although PVP doping reversed this trend (Figure 6(b)). Notably, the PVA-doped SWCNTs exhibited an n-type behavior, contrary to an earlier report of a p-type behavior by Dettlaff – Weglikowska [150], suggesting that factors such as the doping concentration and SWCNT diameter also play critical roles.

Zhang et al. reported a unique increase in both the electron conductivity and absolute Seebeck coefficient in PVP-coated SWCNTs, contrary to the typical trade-off in conventional semiconductors (Figure 6(c)) [153]. A PVP-coated SWCNT sheet synthesized at 483 K with a SWCNT/PVP mass ratio of 1/3 exhibited an electrical conductivity of 905 S cm^−1^ at 307 K, which was significantly higher than that of the pristine SWCNT sheet (~215 S cm^−1^), indicating effective carrier doping from the polymer (Figure 6(d)). Furthermore, for an SWCNT/PVP composite film with a mass ratio of 1/2, increasing the coating temperature changed the doping behavior from *p*- to n-type (Figure 6(e)), suggesting a combined thermodynamic and intrinsic doping mechanism involving hydrogen bonding between the PVP and SWCNT defect sites (Figure 6(f)).

In contrast to polymer/CNT composites, in which CNTs are embedded in a polymer matrix, the amount of polymer coating is crucial for determining the doping efficiency, especially for n-type doping. This amount could be controlled by varying the concentration of the polymer solution. Zhou et al. systematically studied the concentration dependence of PEI and found that p-type SWCNTs doped with 0.01 wt% PEI became n-type when doped with 0.05 wt% PEI (Figure 6(g,h)) [154]. The absolute value of the Seebeck coefficient increased for concentrations up to 0.5 wt% but decreased beyond that concentration.

Wang et al. discovered a solvent-dependent doping effect in polyaniline (PANI)-coated SWCNTs (Figure 6(i)) [155]. Whereas water-based coatings resulted in a p-type behavior, as previously reported [156–168], switching the solvent to DMSO and applying sonication led to an n-type behavior and a higher Seebeck coefficient. They attributed this to the formation of leucoemeraldine base (PANI-lb) (Figure 6(j)), which has a higher HOMO level than emeraldine base (PANI-eb), forming a more crystalline coating on the SWCNTs under sonication treatment, and effectively donating electrons while blocking oxygen adsorption (Figure 6(k)).

#### 2.2.2. Oxygen blocking

**Figure 7.**
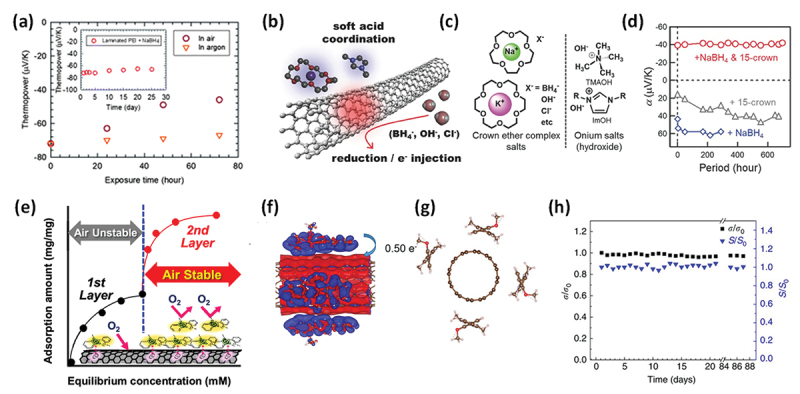
(a) Changes in Seebeck coefficient for PEI-coated SWCNT films in air (red), in argon (orange), and under lamination (inset). Reproduced by permission from [170], copyright 2012, royal society of chemistry. (b, c) schematic concept of salt-induced n-type doping (b) and the typical n-dopant structure (c). (d) Temporal changes in the Seebeck coefficient of SWCNT films treated with NaBH_4_, 15-crown-5-ether, and both over a month under ambient conditions. Reproduced by permission from [171], copyright 2016, wiley-VCH GmbH. (e) Adsorption isotherm of DMBI on SWCNT films. Air stable concentrations overwrapped with the 1^st^ layer adsorption. (f, g) electron density difference map (f) and results of Bader population analysis (g) for four *o*-MeO – DMBI on SWCNTs. Reproduced by permission from [172], copyright 2019, American chemical society. (h) Changes in Seebeck coefficient and electrical conductivity of PEI-coated SWCNTs doped by 1.0 wt% PEI. Reproduced by permission from [154], copyright 2017, Springer nature limited.

It is well known that n-doped CNTs are unstable upon exposure to air, primarily as a result of oxidation by O_2_ or H_2_O [169]. Yu et al. was the first to report that the n-type behavior of n-doped SWCNTs degraded significantly upon exposure to ambient conditions in PEI-coated SWCNT films [170]. They also found that encapsulating the films with polyester sheets could preserve their n-type properties for more than 25 days (Figure 7(a)). Nonetheless, for TEG applications, encapsulating such thermally insulating films hinders their responsiveness to temperature gradients.

In a pioneering work, Nonoguchi et al. developed air-stable n-type SWCNTs using small-molecule doping with salt anions (Figure 7(b,c)), such as Cl^−^, BH_4_^−^, and OH^−^, with counter cations stabilized by tetraalkylammonium (R_4_N^+^) or crown ethers. They highlighted the critical importance of the counter cation size in compensating for the negatively charged SWCNTs and achieving air stability for over 600 h (Figure 7(d)) [171]. Furthermore, it was demonstrated that the dopant coverage of the SWCNT surface was crucial for air stability [172]. In the case of dimethylbenzimidazole-based (DMBI)-based doping, the adsorption isotherms revealed that the n-type SWCNTs achieved long-term air stability only when the DMBI coverage exceeded that of a monolayer (Figure 7(e)). Because the dopant layers become positively charged owing to electron transfer, these cationic layers effectively suppress attack from O_2_ and prevent oxidation (Figure 7(f,g)). This mechanism explains the discrepancy between the results reported by Yu et al. [170] and Zhou et al. [154] concerning the stability of PEI-doped SWCNTs. Zhou et al. examined the effect of the PEI solution concentration and achieved long-term air stability when using a 1 wt% PEI solution, whereas Yu et al. did not investigate the concentration dependence. For polymer doping, the concentration of the dopant solution significantly affects both the carrier type and air stability because it determines the surface coverage of the SWCNTs. These findings underscore the importance of the coating morphology in achieving stable polymer-based doping.

#### 2.2.3. Energy filtering

A polymer coating layer on CNTs can raise the Seebeck coefficient relative to uncoated films via an interface-driven mechanism at CNT/polymer/CNT junctions. An interfacial potential offset preferentially impedes low-energy carriers, increasing the average carrier energy and thereby enhancing the Seebeck coefficient, often with only a modest impact on electrical conductivity [173]. The term ‘energy filtering’ was originally introduced in thermoelectric theory as a k-space phenomenon. To avoid conflation with this distinct concept, we refer to the real-space heterointerface effect discussed here as energy-dependent barrier scattering (or barrier scattering). This energy-dependent carrier scattering process can lead to significant improvements in the power factor ( =S^2^). Theoretical studies using silicon revealed that the energy offset should be optimized to <0.2 eV and the gap of the heterojunction should be controlled at ~3 nm.

**Figure 8.**
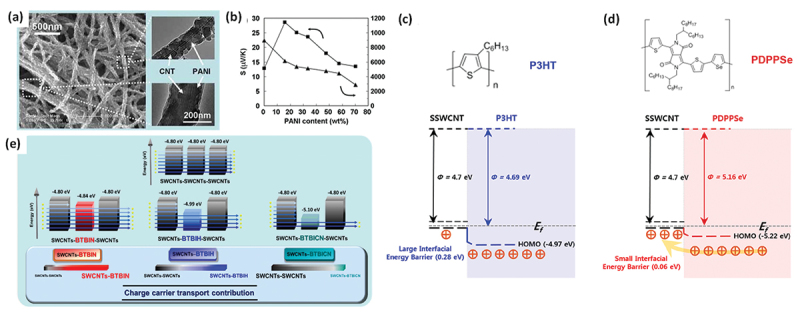
(a) SEM images of PANI-coated CNT sheets prepared with different aniline concentrations. (b) Seebeck coefficient and electrical conductivity of PANI-coated CNT sheet as a function of PANI content at 300 K. Reproduced by permission from [174], copyright 2010, wiley-VCH GmbH. (c, d) equilibrium band diagrams for the SWCNT/P3HT (c) and SWCNT/PDPPSe interfaces (d). Reproduced by permission from [175], copyright 2019, American chemical society.(e) schematic illustration of energy filtering in SWCNT−SOM hybrids and contribution of SWCNT−SOM−SWCNT charge carrier transport in hybrids compared to that of SWCNTs−SWCNTs−SWCNTs. Reproduced by permission from [176], copyright 2025, American chemical society.

In 2010, Fan et al. first reported the energy-dependent carrier scattering of CNTs using PANI-coated MWCNTs prepared via the electrochemical polymerization of aniline onto an MWCNT network (Figure 8(a)) [174]. The coating thickness, and thus the gap distance, of the CNT/PANI/CNT heterojunctions could be tuned by adjusting the polymerization time. They found that the power factor increased significantly with an optimal PANI content of 10 wt%, indicating effective energy-dependent carrier scattering by the thin PANI layer. The PANI-coated MWCNT film exhibited a power factor that was nearly four times that of the pristine MWCNTs and 10^4^ times that of PANI alone. Additionally, the thermal conductivity was reduced by an order of magnitude, both of which improved the thermoelectric figure of merit (zT).

A critical requirement for effective energy-dependent carrier scattering in polymer/SWCNT heterointerfaces is a small work function difference. For example, in 2019, Cho et al. observed efficient energy filtering in poly (diketopyrrolopyrrole-selenophene) (PDPPSe)/SWCNT composites, with a work function difference of only 0.06 eV. In contrast, the larger difference (0.28 eV) found in P3HT/SWCNT composites did not yield this effect (Figure 8(c,d)) [175]. A similar effect was also reported for polymer-coated CNT films, polymer/CNT composites, and small-molecule doping systems. Hong et al. reported that a low energy barrier (0.04 eV) between the SWCNT valence band and HOMO level of small-molecule dopants facilitated interfacial energy-dependent carrier scattering, whereas a larger barrier (0.30 eV) had adverse effects (Figure 8(e)) [176].

Although an increase in the Seebeck coefficient often comes at the cost of reduced electrical conductivity, conjugated polymers such as PEDOT:PSS [177–179], polyaniline [180,181], polypyrrole [182], and Schiff base polymers [183] have been investigated because of their potential to achieve synergistic effects in polymer-coated CNTs and their composites.

#### 2.2.4. Extraction of semiconducting CNTs

For SWCNT-based thermoelectric devices, the use of sc-SWCNTs is desirable because of their intrinsically high Seebeck coefficients [184–190], especially those with small diameters [191]. In this regard, PFO derivatives represent a unique and powerful class of polymers capable of selectively extracting sc-SWCNTs from mixtures of semiconducting and metallic SWCNTs with high purity (~99%) in aromatic solvents such as toluene. This technique was first reported by Nish et al. [31] Owing to the ease of processing and tunability of the chemical structure of PFO, numerous derivatives have been developed that allow the efficient dispersion and centrifuge-based extraction of sc-SWCNTs [192–202]. Among them, PFO with a bipyridine (BPy) moiety (PFO-BPy) has been the most widely employed for selectively isolating (6,5) sc-SWCNTs with a diameter of 0.76 nm.

Despite their promising thermoelectric properties, sc-SWCNTs suffer from a low intrinsic electrical conductivity and cannot be effectively doped with PFO alone. Therefore, systematic doping studies using chemical oxidants and reductants have been conducted on PFO-coated sc-SWCNTs [185,186,203]. Because these PFO-coated films maintain characteristic interband transitions such as the S11 band, their doping states can be quantitatively evaluated using optical spectroscopy.

**Figure 9.**
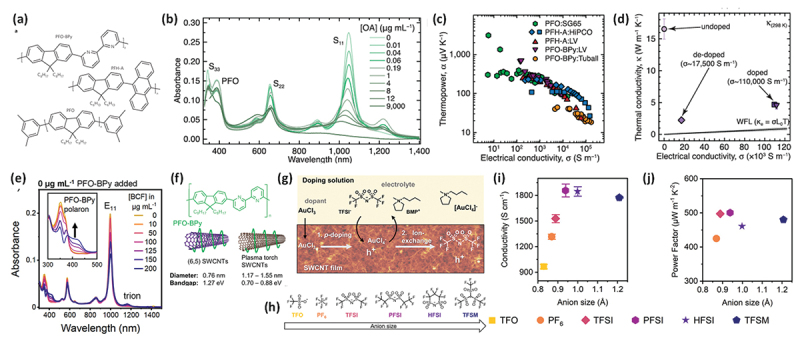
(a) Chemical structures of PFOs (PFO-Bpy, PFH-A, and PFO). (b) Change in absorption spectra of PFO-coated SWCNTs upon OA doping. (c) Plot of Seebeck coefficient as a function of the electrical conductivity of PFO-coated SWCNT sheets. (d) Thermal conductivity of a PFO-BPy-coated SWCNT thin film as a function of the electrical conductivity near 300 K. Reproduced by permission from [185], copyright 2016, Springer nature limited. (e) Normalized E11 bleach values for both dispersions depending on the bcf concentration. Reproduced by permission from [204], copyright 2024, American chemical society. (f) Chemical structure of PFO-BPy and schematics of the PFO-BPy-coated (6,5) SWCNTs (large bandgap) and plasma torch SWCNTs. (g) Ion-exchange doping reaction scheme for a SWCNT film doped by AuCl_3_ and anions exchanged with [BMP][TFSI]. (h) Molecular structures of anions of different sizes used for ion-exchange. Corresponding (i) electrical conductivity, and (j) power factor of (6,5) SWCNT films versus anion size. Reproduced by permission from [205], copyright 2024, wiley-VCH GmbH.

Avery et al. and Norton-Baker et al. doped PFO-coated sc-SWCNTs (~1.3 nm) with oleylamine (OA) and monitored the doping state using NIR absorption spectroscopy in conjunction with Seebeck coefficient and electrical conductivity measurements (Figure 9(a–c)) [185]. They achieved a maximum power factor of approximately 340 µW m^−1^ K^−2^ for PFH-A-coated HiPco films.

Zaumseil et al. investigated the p-doping of PFO-BPy-coated SWCNTs with tris (pentafluorophenyl)borane (BCF). Notably, doping did not affect the solubility of the PFO-BPy-coated SWCNTs, and absorption spectroscopy revealed the bleaching of the S11 band, as well as the formation of PFO-BPy polarons and SWCNT trions (Figure 9(e)) [204]. These observations, supported by NMR measurements, indicated a doping mechanism wherein BCF forms a complex with the BPy moiety, leading to electron transfer from the SWCNTs to the polymer, resulting in hole doping. They also explored the effect of anion size by doping PFO-BPy-coated sc-SWCNTs with AuCl_3_ followed by the ion exchange of Cl^−^ with larger anions (Figure 9(f–h)) [205]. When bis(trifluoromethanesulfonyl)imide (TFSI^−^) was incorporated, the power factor exceeded 500 µW m^−1^ K^−2^. Molecular simulations revealed that larger anions promoted the formation of impurity bands near the valence band edge, facilitating interband hopping and enhancing the electrical conductivity. However, the increased charge separation between the SWCNTs and anions led to shallower impurity bands and a narrower one-dimensional density of states, thereby reducing the Seebeck coefficient. This insight offers new guidelines for the rational design of dopants.

Avery et al. also discovered that chemical doping significantly reduced the in-plane thermal conductivity of PFO-coated sc-SWCNT films – from ~16.5 W m^−1^ K^−1^ to 4.5 W m^−1^ K^−1^ —while increasing the electrical conductivity from nearly zero to 110,000 S m^− 1^ [184]. This led to a synergistic enhancement in the thermoelectric figure of merit, zT = S^2^ σT/κ, where κ represents the thermal conductivity. Typically, the thermal conductivity of CNT sheets is two orders of magnitude lower than that of isolated CNTs ( >1000 W m^−1^ K^−1^) because of phonon scattering at inter-tube junctions. When polymers are introduced, vibrational and mechanical mismatches at the polymer/CNT interfaces or CNT/polymer/CNT heterojunctions further increase the thermal impedance, thereby lowering κ [175,206–211]. For PFO-BPy-coated sc-SWCNTs, a pristine film exhibited negligible electrical conductivity (σ ≈ 0 S m^−1^), which indicated that the thermal transport was dominated by lattice thermal conductivity (κ_e_), with minimal contribution from electronic conductivity (κₑ). Importantly, the sharp reduction in κ after doping confirmed that the thermal conductivity was largely decoupled from σ.

This result suggests that (1) the thermal and electrical conductivities can be independently controlled in SWCNTs and (2) dopant molecules or morphological changes introduced via doping can reduce the phonon mean free paths, thereby enabling a synergistic zT enhancement. However, in most cases, defect-induced phonon scattering also leads to carrier scattering, which reduces the electrical conductivity. Therefore, selective phonon scattering using engineered heterointerfaces remains a major challenge.

#### 2.2.5. Challenges for polymer coating of TE materials

In SWCNT network films, the main source of electrical resistance is the contact resistance at the CNT junctions, a phenomenon that is also observed in thermal transport. This contact resistance is further exacerbated in polymer-coated systems, regardless of the electrical conductivity of the polymer, often resulting in a reduced electrical conductivity compared to that of the uncoated counterparts, which in turn can degrade the overall thermoelectric performance [212].

**Figure 10.**
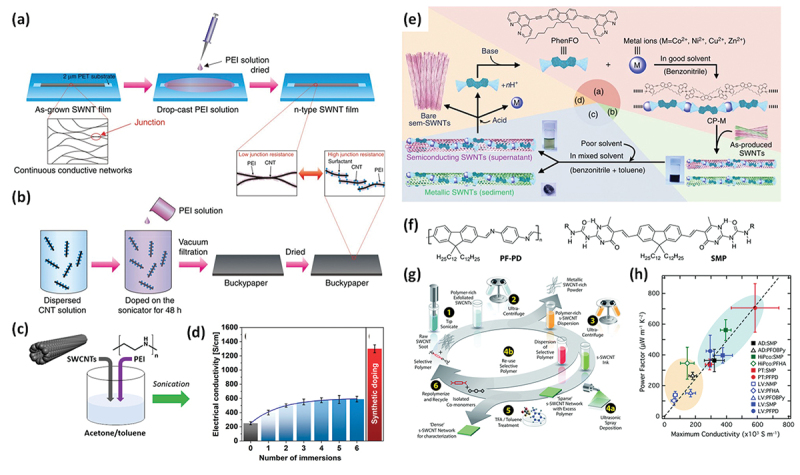
(a, b) schematics of the fabrication processes for (a) n-doped SWCNT films based on as-grown SWCNT continuous networks and (b) n-doped SWCNT films based on dopant-dispersed SWCNT solutions. Reproduced by permission from [154], copyright 2017, Springer nature limited. (c) Schematic of PEI doping of SWCNTs by coating. (d) Electrical conductivities of SWCNT films fabricated using sequential doping (blue bars) and a PEI solution (red bar). Reproduced by permission from [213], copyright 2020, MDPI. (e) Method for sc-SWCNT sorting using removable dispersant, starting from the preparation of coordination polymer ligated with metals (CP-M) (*M* = Co, Ni, Cu, and Zn) to the dispersion of the as-produced SWCNTs, separation of s- and m-SWCNTs, and removal and recovery of the adsorbents. Reproduced by permission from [201], copyright 2014, Springer nature limited. (f) Chemical structures of cleavable polymers. (g) Generalized process for making high-performance sc-SWCNT thin films with no residual sorting polymer. (h) The maximum conductivity and power factor for all networks utilizing cleavable polymers (blue oval) exceed the values for networks containing residual wrapping polymer (orange oval). Reproduced by permission from [214], copyright 2017, the royal society of chemistry.

One approach to mitigate this issue is ‘sequential doping’, in which the pristine SWCNT network film is first fabricated and then coated with the dopant polymer (Figure 10(a)), rather than doping the SWCNTs prior to film formation (Figure 10(b)) [154]. This strategy preserves the direct SWCNT–SWCNT contact at the junctions, maintaining the high electrical conductivity of the original network. However, the efficiency of sequential doping depends on the sufficient diffusion of the polymer into the SWCNT network, which can be limited in thicker or densely packed films.

Rdest et al. compared PEI-coated SWCNT films fabricated using PEI-dispersed solutions (Figure 10(c)) with those produced via sequential doping. They found that a film prepared using a PEI solution a exhibited significantly higher electrical conductivity (1301 ± 56 S cm^−1^) than those treated by sequential doping (593 ± 21 S cm^−1^) (Figure 10(d)) [213]. This result suggests that in sequential doping, the insufficient diffusion of PEI into the SWCNT network leads to inadequate coating and ineffective doping.

Another strategy for addressing contact-resistance issues involves the use of removable polymers. Inspired by the pioneering work of Toshimitsu et al. (Figure 10(e)) [201], MacLeod et al. developed imine-based and hydrogen-bonded PFOs for TE applications, both of which could be removed through acid treatment (Figure 10(f)) [186]. Upon polymer removal, significant improvements in electrical conductivity were observed (Figure 10(g)). These removable PFO-coated sc-SWCNT films (with a semiconducting purity of >99% and trace amount of PFO) achieved a high power factors of ~705 µW m^−1^ K^−2^ and zT values of ~0.12. Although these values were still lower than those of the optimized bismuth telluride, which had a zT value of ~0.6, the inherent advantages of CNT-based materials, such as flexibility and light weight, continue to drive interest in their further development. Notably, the same removable-PFO approach has been successfully applied in the development of thin-film transistors [214].

### 2.3. Electrochemical devices

CNTs are considered promising electrode materials for electrochemical applications such as batteries and capacitors, owing to their mechanical strength, exceptional electrical conductivity and electrochemical stability [215]. In particular, there is a significant demand to improve the durability of polymer electrolyte membrane fuel cells (PEMFCs) [215]. This has led to the adoption of CNTs as electrically conductive support materials for metal catalysts, replacing conventional carbon supports such as carbon Black, which suffer from lower crystallinity.

#### 2.3.1. Metal loading

CNTs have often been investigated as electrodes for various electrochemical devices, especially PEMFCs, where the exceptional electrical conductivity and electrochemical stability of CNTs are desirable.

**Figure 11.**
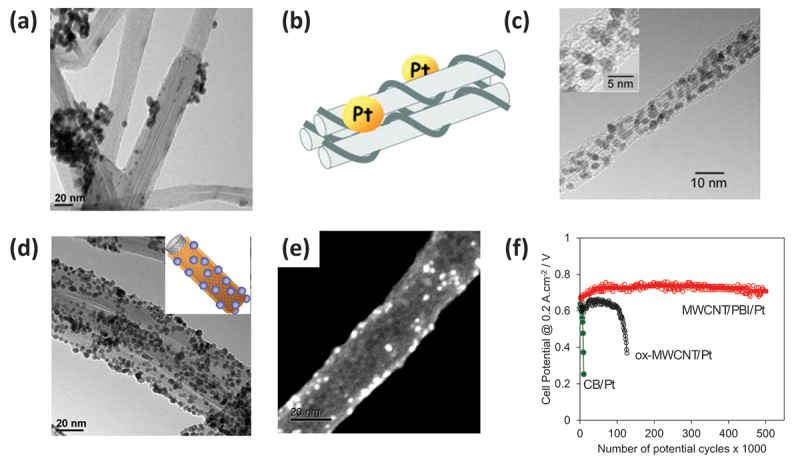
(a) TEM image of Pt-loaded MWCNTs. Reproduced by permission from [216], copyright 2009, American chemical society. (b, c) illustration of Pt-loaded PSS-coated SWCNTs (b) and their TEM image (c). Reproduced by permission from [219], copyright 2006, American chemical society. (d, e) TEM image of Pt-loaded PBI-coated MWCNTs (inset: illustration of Pt-loaded PBI-coated MWCNTs) (d) and their SEM image (e). Reproduced by permission from [216] for (d) and from [50] for (e), copyright 2009, American chemical society and copyright 2014, Springer nature limited, respectively. (f) Plot of cell voltages obtained in durability tests of single cells containing conventional Pt catalyst loaded on CB (green), oxidized MWCNTs (Black), and PBI-coated MWCNTs (red). Reproduced by permission from [221], copyright 2015, Springer nature limited.

However, a major drawback of using highly crystalline carbon materials such as CNTs is the difficulty in achieving a homogeneous loading of metal nanoparticles on their surfaces owing to the absence of anchoring sites (Figure 11(a)) [216]. To address this issue, the surface oxidation of CNTs has been commonly employed to introduce anchoring sites. However, this process also results in the formation of sp^3^ carbon [217,218] and often involves a loss of electrochemical stability. In this regard, polymer-coated CNT contributes useful material that can keep intactness of CNT while offering anchoring site for catalyst loading. Kamat et al. reported the deposition of Pt nanoparticles onto polystyrene sulfonate (PSS)-coated CNTs for use as electrocatalysts for methanol oxidation (Figure 11(b,c)) [219]. Although their work focused on debundling the SWCNTs and the measurement was limited to half-cell experiments, it offered valuable insights into the use of polymer coatings for metal loading without compromising the integrity of the CNT surface. It is important to note that that polymer coating layer for polymer-coating CNTs is typically thin enough to allow electronic communication between CNT and catalysts through tunneling.

Building on this concept, we used PBI as coating polymer and successfully loaded Pt nanoparticle on MWCNTs (Figure 11(d)) [216]. PBI was selected because of its chemical and thermal stabilities, ability to form a stable coating on CNTs, and nitrogen-containing groups suitable for metal ion coordination. Pt nanoparticles were deposited via a conventional polyol method using ethylene glycol (EG) as both the solvent and reducing agent, and a Pt salt as the precursor (Figure 11(d)) [216,220]. The quantitative deposition of Pt ions was achieved on PBI-coated CNTs without introducing sp^3^ defects [216,220]. The coordination between the Pt ions and PBI was confirmed by IR spectroscopy (Pt–N band at 560 cm^−1^) and N 1s XPS analysis [220]. As explained in Mechanism of adsorption-based polymer coating, the real coverage of the PBI coating on the carbon materials was incomplete, as simplified in the illustration (inset in Figure 11(d)). Such partial coverage was successfully visualized in an SEM image, where the exposed CNT surface is seen in the darkest area, and the covered area is gray in color (Figure 11(e)) [50]. The White spots in this image are Pt nanoparticles. The image clearly shows that the Pt nanoparticles were immobilized on the PBI-covered area and not on the bare CNT surface. Importantly, we demonstrated that PEMFCs employing PBI-coated MWCNTs had a significantly greater durability than those using oxidized MWCNTs (Figure 11(f)) [221,222].

PBI-coated CNTs have also been used to load other metals, such as Au [223,224], Pd [225], Ir [226], Cu [227], and Ni-Co [228] nanoparticles, for a variety of electrochemical devices including water electrolyzers [226], CO_2_ reduction cells [224,229], and oxygen evolution electrodes [228]. Many research groups have employed PBI-coated CNTs for nanoparticle immobilization [230–240]. For example, Yang et al. deposited anatase TiO_2_ on PBI-coated MWCNTs for the electrosynthesis of glycolic acid [240]. Hu et al. immobilized Ag nanoparticles on PBI-coated MWCNT sheets to create reusable catalysts for 4-nitrophenol reduction [238]. Tominaga et al. deposited cobalt oxide on PBI-coated MWCNTs and developed a highly sensitive phosphate sensor [230]. Kato et al. reported a higher oxygen reduction reaction (ORR) activity for Pt–Ni nanoparticles supported on PBI-coated CNTs compared to uncoated CNTs [235]. Importantly, they found that the PBI layer did not induce electronic perturbations in the metal catalysts, unlike nitrogen-doped carbon, which often alters the electronic structure of the catalysts.

In addition to PBI, other polymers, such as polyimide [241] and PVP [242] have also been used for polymer-assisted metal loading. One unique example involves PVP-coated CNTs and graphene, which were decollated using metal – organic frameworks (MOFs) and subsequently employed for CO_2_ capture [243]. A particularly successful example of a nanoscale polymer coating is the use of polyimide on lithium titanate anodes in rechargeable LIBs [244]. These coatings effectively inhibited side reactions with the electrolytes, improving both the cycling stability and rate performance.

#### 2.3.2. Ionomer adsorption layer

Polymer coatings have also been used to improve the performances of PEMFCs through facilitating the cathode reaction (O_2_ +4 H^+^ +4e^−^→2 H_2_O). In PEMFCs, optimizing the ionomer distribution in the cathode catalyst layer is essential for maximizing the activity and stability because it strongly affects the diffusion of both protons and oxygen, where a thin and homogeneous coating is preferable. When PBI-coated CNTs were used as conductive support, strong adsorption of the ionomer onto the PBI layer was observed. This strong interaction contributed to the formation of a thin and homogeneous ionomer distribution within the catalyst layer. In addition, such strong interactions helped prevent ionomer leaching, thereby improving the durability of the PEMFCs. This effect was confirmed using poly(vinylphosphonic acid) (PVPA) and Nafion ionomers [245]. Controlling the wettability of the catalyst layer remains a major challenge in the design of PEMFC cathodes. Polymer coatings offer a promising alternative to covalent surface modification, which often reduces electrochemical stability.

**Figure 12.**
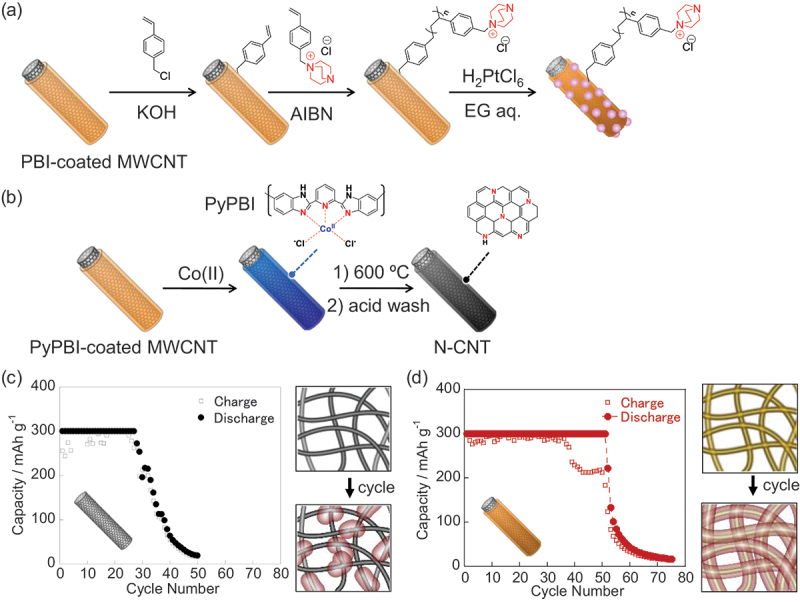
(a) Schematic illustration of the grafting of DABCO cations onto PBI-coated MWCNT. (b) Schematic illustration of nitrogen-doped carbon formation on MWCNT to form N-CNT. (c, d) cycling stability values of Li-air batteries using SWCNTs (c) and PyPBI-coated SWCNTs (d) as cathodes (1 mg/cm^2^), in which TEGDME containing 1.0 M LiTFSI was used as the electrolyte, and charge/discharge cycling was carried out at 0.1 mA cm^−1^. The diagrams indicate the deposition of Li oxide on the cathodes upon charge/discharge cycling, in which the Li oxide was deposited inhomogeneously on the SWCNT cathode (c) but homogeneously on the PyPBI-coated SWCNT cathode (d). Reproduced by permission from [250], copyright 2019, Springer nature limited.

In addition to physical adsorption, the chemical grafting of ionomers onto PBI-coated CNTs is also possible, leveraging the reactivity of the imidazole groups in PBI. We successfully introduced quaternized 1,4-diazabicyclo[2.2.2]octane (DABCO) onto PBI-coated MWCNTs (Figure 12(a)) and applied them in anion exchange membrane fuel cells without the need for additional ionomer components [246]. This study demonstrated the polymer coating layer can act not only for catalyst anchoring sites but as ion conduction pathways when properly designed.

An interesting application of PBI-coated CNTs is in the formation of CNTs coated with N-doped graphitic layers. These were formed by heating PBI-coated CNTs to create N-CNTs (Figure 12(b)), which exhibited ORR activity [247–249].

The polymer-coated CNT approach also contributes to wettability control in Li-air (O_2_) rechargeable batteries [250]. In Li-air (O_2_) batteries, lithium ions from the anode react with O_2_ to form insoluble lithium oxides at the cathode during discharging. However, the accumulation of Li_2_O often clogs the electrode pores [251], limiting the charge capacity and rechargeability. We found that using PyPBI-coated SWCNTs as the cathode improved rechargeability and reduced the overpotential by promoting the homogeneous nucleation of lithium oxide through coordination between the PyPBI and Li^+^ ions (Figure 12(c,d)). This resulted in the formation of a uniform Li oxide layer, in contrast to the uneven deposition observed on the uncoated SWCNT cathodes [250]. This versatility in the surface chemistry and functionality makes PBI-coated CNTs valuable platforms for a wide range of electrochemical applications.

#### 2.3.3. Pseudocapacitive layer for supercapacitors

Polymer-coated CNTs employing redox-active conducting polymers (PANI, PPy, PEDOT) are compelling supercapacitor electrodes [54,252,253]. Conformal polymer shells contribute pseudocapacitance via fast, near-surface Faradaic reactions, while intimate contact with CNT scaffolds lowers interfacial resistance and shortens ion-diffusion paths relative to particle – binder architectures, enabling high-rate operation and robust cycling in flexible formats. Electrochemical polymerization provides angstrom-level thickness control and substrate selectivity, permitting binder-free, high-loading deposition of redox-active polymers onto CNT films and fabrics with competitive gravimetric capacitance; PANI-coated CNTs, in particular, have been extensively investigated [254] and systematically reviewed [53,255]. Recent studies emphasize core – shell architectures of polymer-coated CNTs to mitigate polymer swelling/shrinkage, preserve electronic pathways, and suppress contact loss and capacitance fade during long-term cycling – for example, a PANI-coated CNTs composite delivered ~354 F g^−1^ at 0.5 A g^−1^ with ~88% retention after 5000 cycles [256], attributed to the conductive CNT core and mechanically stabilized PANI shell. Further enhancements in mechanical robustness and ion transport for flexible supercapacitors have been achieved by crosslinking of polymer layers and by hybridizing CNTs with two-dimensional carbons or MXenes [255,257–259].

### 2.4. Biological applications

**Figure 13.**
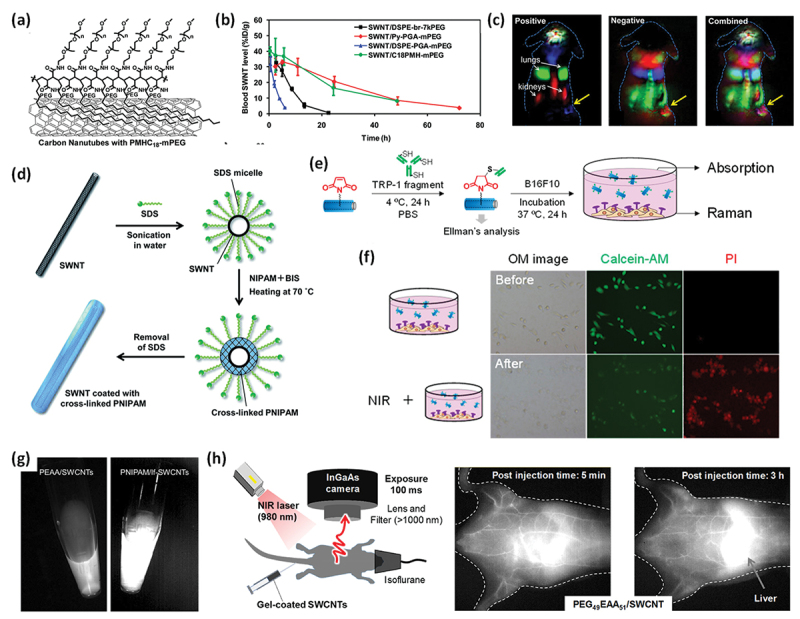
(a, b) structures of PMHC_18_-mPEG-coated SWCNTs (a) and their blood circulation curves (green) (b). Reproduced by permission from [265], copyright 2009, American chemical society. (c) Dynamic contrast-enhanced images based on PCA analysis of NIR-II fluorescence images of a tumor-bearing mouse after injection of PMHC_18_-mPEG-coated SWCNTs: (left panel) positive pixels from PCA, showing lungs, kidneys, and major vessels in the tumor; (center panel) negative pixels from PCA, showing the body of the tumor; (right panel) overlaid image showing the absolute values of both positive and negative pixels, from which both the vessels in the tumor and the tumor outline can be seen. The yellow arrows in the images highlight the tumor. Reproduced by permission from [266], copyright 2012, American chemical society. (d) Schematic illustration of CNT micelle polymerization. Reproduced by permission from [268], copyright 2014, the royal society of chemistry. (e, f) anti-body conjugation of maleimide-containing gel-coated SWCNTs and their specific binding of target cells (e), along with the death of the target cells triggered by NIR-irradiation (f). Reproduced by permission from [272], copyright 2021, American chemical society. (g) Comparison of the brightness levels of NIR emissions for SWCNTs without (left) and with (right) quantum defects. Reproduced by permission from [274], copyright 2020, American chemical society. (h) NIR imaging set-up for mice and their NIR-II images after injecting gel-coated SWCNTs. Reproduced by permission from [275], copyright 2024, Elsevier.

SWCNTs are promising materials for *in vivo* applications because of their unique NIR absorption and emission characteristics. For such biomedical applications, a stable and biocompatible dispersion of SWCNTs is essential, similar to that of other nanomaterials. The covalent modification of SWCNTs often deteriorates their optical properties in the NIR region; thus, non-covalent dispersions using surfactants are commonly employed. In particular, biological surfactants such as polyethylene glycol (PEG)-based phospholipids (PL-PEG) are often used, and many successful in vivo examples have been reported [10,260–263]. In 2008, Liu et al. reported an increase in the circulation time for blood as the molecular weight of the PEG unit increased, owing to the higher coverage of the SWCNT surface, which prevented opsonization and accumulation in reticuloendothelial organs [264]. Although this system utilizes dynamic adsorption on a CNT surface based on amphiphilic molecules, it provides clear guidance for designing polymer coating systems with greater stability. In 2009, Prencipe et al. developed PEG-grafted poly(maleic anhydride-alt-1-octadecene) (PMHC_18_-mPEG) as a novel coating polymer for SWCNTs (Figure 13(a)) and found that it increased the circulation time for blood compared to that with PL-PEG (Figure 13(b)) [265]. They successfully monitored the distribution of SWCNTs using NIR fluorescence and found that PMHC_18_-mPEG-coated SWCNTs accumulated in tumors via the enhanced permeation retention (EPR) effect (Figure 13(c)) [266]. In addition to PEG-based amphiphilic polymers, studies have been conducted on amphiphilic polymers with betaine moieties [267].

We developed a novel coating method for SWCNTs coated with cross-linked polymers (i.e. hydrogels), which was achieved by the radical polymerization of monomers in the presence of cross-linkers and initiators using surfactant-dispersed SWCNTs (Figure 13(d)) [268]. During polymerization (CNT micelle polymerization, CMP), oligomers migrate into the micelle interior, where crosslinking and polymer growth occur on the CNT surface, forming a thin gel layer that remains intact even after surfactant removal. Unlike conventional polymer coating approaches that rely on the adsorption of a polymer onto SWCNT surfaces based on its amphiphilicity, micelle-mediated polymerization does not require a polymer with amphiphilicity. This coating relies on the hydrophobicity of monomers or their oligomers, similar to soap polymerization. Therefore, a wide range of vinyl monomers can be incorporated, and monomers such as *N*-isopropylacrylamide (NIPAM), methyl methacrylate (MMA), and poly(ethylene glycol) methacrylate (PEGMA) have been successfully incorporated to form a gel-coating layer on SWCNT surfaces [269]. When PEGMA was used as a comonomer, the resulting PEGylated gel coatings exhibited excellent dispersibility in water, buffered solutions, and serum [270]. These coatings were nearly charge-neutral and their stability was attributed to the excluded volume effect of the PEG chains. Moreover, we synthesized a vinyl monomer containing a maleimide group and successfully incorporated it into a gel layer, which enabled the post-functionalization of gel-coated SWCNTs via ene-thiol chemistry [271]. Such post-functionalization has not yet been achieved for polymer-coated SWCNTs. Using this functionalized coating, antibodies were conjugated to the SWCNTs, and the specific targeting of cancer cells was demonstrated in vitro (Figure 13(e)) [272]. Upon NIR irradiation, the targeted cancer cells were effectively ablated via photothermal conversion (Figure 13(f)).

In a related study, Numata et al. conjugated DNA onto gel-coated SWCNTs using mitochondria-targeting peptides, which enabled the successful delivery of DNA into the mitochondria of plant cells [273]. Furthermore, we discovered that the polymerization process can introduce quantum-defect-localized sites that emit photoluminescence (PL) at wavelengths longer than the original E_11_ emission by controlling the amount of initiator (Figure 13(g)) [274]. Without quantum defects, the S_22_ transition, which is typically in the visible range, must be used as the excitation source, whereas the S_11_ transition, which is typically in the NIR-I or NIR-II range, can be used with quantum defects. Consequently, NIR-II excitation with NIR-II emission was possible, which is preferable for bioimaging. Indeed, these bright NIR-II emissions ( > 1100 nm) enabled us to visualize blood circulation in vivo at low doses. Based on this emission, we determined that the gel-coated SWCNTs remained in circulation for over 24 h in mice (Figure 13(h)) [275].

Owing to their robust surface stability, gel-coated SWCNTs are compatible with length-separation techniques based on gel permeation chromatography using water or buffer solutions as eluents [276]. Such chromatographic separation has only been reported for DNA-coated SWCNTs because of their stable coating [27], whereas the addition of a surfactant to the eluent is necessary for surfactant-dispersed SWCNTs to maintain the dynamic replacement of surfactants [277]. The length fractionation of gel-coated SWCNTs allowed for systematic length-dependency studies in vivo. Based on the above-mentioned functions, gel-coated SWCNTs are recognized as a versatile platform for nanobioapplications and have been used as ideal NIR emitters for bioimaging [278].

## 3. Summary

Polymer coatings have emerged as an effective approach for tailoring the surface chemistry and interfacial functionality of CNTs, while preserving their intrinsic electronic and mechanical properties. This review provided a comprehensive overview of polymer-coated CNTs, focusing on the coating mechanisms, interfacial interactions, and resulting structure – property relationships. Emphasis was placed on how polymer wrapping and adsorption influence the dispersion stability, surface energy modulation, and charge transfer between the CNTs and surrounding matrices.

This review highlighted the recent progress in applications across multiple fields, including sensing, energy conversion, catalysis, and biomedicine. Polymer-coated CNTs enable selective molecular recognition and improve the signal transduction in gas and ion sensors. In thermoelectric systems, the polymer layers contribute to energy filtering, environmental stability, and tunable carrier control. In electrochemical energy devices, polymer coatings facilitate uniform catalyst dispersion and ion-transport regulation. Furthermore, biocompatible polymer and hydrogel coatings have expanded the utility of CNTs in imaging, therapy, and controlled drug delivery.

In this review, we mainly focused on the PBI-coated CNTs, however, there are still many exploring spaces to develop new polymer-coated CNT when considering wide range of polymer choice. Therefore, future research is expected to leverage data-driven approaches, such as machine learning using monomer descriptors to predict target properties of polymer-coated CNTs such as conductivity, selectivity, Seebeck coefficient, biocompatibility and so on, thereby accelerating the development of next-generation functional CNT materials.

At the same time, fundamental studies to understand adsorption morphology such as coverage and orientation of polymers exist as challenges to tackle and advanced instrumental analysis techniques such as AFM based spectroscopy or interface spectroscopy is necessary to carry out. We believe that these fundamental studies will be feedback to further enhancement of the performance of polymer-coated CNTs and the same concept can be applicable to the other carbon materials such as graphene, carbon Black, carbon fibers and so on.

## References

[1] Krueger A. Carbon materials and nanotechnology. Weinheim, Germany: Wiley-VCH Verlag GmbH & Co. KGaA; 2010.

[2] Bolisetty S, Peydayesh M, Mezzenga R. Sustainable technologies for water purification from heavy metals: review and analysis. Chem Soc Rev. 2019;48(2):463–31. doi: 10.1039/C8CS00493E 30603760

[3] Georgakilas V, Perman JA, Tucek J, et al. Broad family of carbon nanoallotropes: classification, chemistry, and applications of fullerenes, carbon dots, nanotubes, graphene, nanodiamonds, and combined superstructures. Chem Rev. 2015;115(11):4744–4822. doi: 10.1021/cr500304f 26012488

[4] Robertson CG, Hardman NJ. Nature of carbon Black reinforcement of rubber: perspective on the original polymer nanocomposite. Polymers. 2021;13(4):538. doi: 10.3390/polym13040538 33673094 PMC7917815

[5] Małecka M, Ciach A, Terzyk AP, et al. Only-sp2 nanocarbon superhydrophobic materials – synthesis and mechanisms of high-performance. Adv Colloid Interface Sci. 2024;334:103311. doi: 10.1016/j.cis.2024.103311 39442424

[6] Iijima S. Helical microtubules of graphitic carbon. Nature. 1991;354(6348):56–58. doi: 10.1038/354056a0

[7] Rathinavel S, Priyadharshini K, Panda D. A review on carbon nanotube: an overview of synthesis, properties, functionalization, characterization, and the application. Mater Sci Eng: B. 2021;268:115095. doi: 10.1016/j.mseb.2021.115095

[8] Wang C, Xia K, Wang H, et al. Advanced carbon for flexible and wearable electronics. Adv Mater. 2019;31(9):1801072. doi: 10.1002/adma.201801072 30300444

[9] Lawal AT. Recent application of carbon nanotubes in energy storage and conversion devices. Carbon Trends. 2025;19:100470. doi: 10.1016/j.cartre.2025.100470

[10] Hong G, Diao S, Antaris AL, et al. Carbon nanomaterials for biological imaging and nanomedicinal therapy. Chem Rev. 2015;115(19):10816–10906. doi: 10.1021/acs.chemrev.5b00008 25997028

[11] Jorio A, Dresselhaus G, Dresselhaus MS. Carbon nanotubes: advanced topics in the synthesis, structure, properties and applications. Heidelberg, Germany: Springer GmbH; 2008.

[12] Mittal G, Dhand V, Rhee KY, et al. A review on carbon nanotubes and graphene as fillers in reinforced polymer nanocomposites. J Ind Eng Chem. 2015;21:11–25. doi: 10.1016/j.jiec.2014.03.022

[13] Welsher K, Sherlock SP, Dai H. Deep-tissue anatomical imaging of mice using carbon nanotube fluorophores in the second near-infrared window. Proc Natl Acad Sci USA. 2011;108(22):8943–8948. doi: 10.1073/pnas.1014501108 21576494 PMC3107273

[14] Nakashima N, Fujigaya T. Fundamentals and applications of soluble carbon nanotubes [Review]. Chem Lett. 2007;36(6):692–697. doi: 10.1246/cl.2007.692

[15] Murakami H, Nakashima N. Soluble carbon nanotubes and their applications. J Nanosci Nanotechnol. 2006;6(1):16–27. doi: 10.1166/jnn.2006.17900 16573065

[16] Nakashima N. Soluble carbon nanotubes: fundamentals and applications. Int J Nanosci. 2005;4(1):119–137. doi: 10.1142/S0219581X05002985

[17] Tasis Nt D, Bianco M, Prato A. Chemistry of carbon nanotubes. Chem Rev. 2006;106(3):1105–1136. doi: 10.1021/cr050569o 16522018

[18] Fujigaya T, Nakashima N. Soluble carbon nanotubes and nanotube-polymer composites [Review]. J Nanosci Nanotechnol. 2012;12(3):1717–1738. doi: 10.1166/jnn.2012.6137 22754974

[19] Skakalova V, Kaiser AB, Dettlaff-Weglikowska U, et al. Effect of chemical treatment on electrical conductivity, infrared absorption, and Raman spectra of single-walled carbon nanotubes. J Phys Chem B. 2005;109(15):7174–7181. doi: 10.1021/jp044741o 16851818

[20] Sun Y-P, Fu K, Lin Y, et al. Functionalized carbon nanotubes: properties and applications. Acc Chem Res. 2002;35(12):1096–1104. doi: 10.1021/ar010160v 12484798

[21] Niyogi S, Hamon MA, Hu H, et al. Chemistry of single-walled carbon nanotubes. Acc Chem Res. 2002;35(12):1105–1113. doi: 10.1021/ar010155r 12484799

[22] Fujigaya T, Nakashima N. Non-covalent polymer wrapping of carbon nanotubes and the role of wrapped polymers as functional dispersants [Review]. Sci Technol Adv Mater. 2015;16(2):024802. doi: 10.1088/1468-6996/16/2/024802 27877763 PMC5036478

[23] Fujigaya T, Nakashima N. Methodology for homogeneous dispersion of single-walled carbon nanotubes by physical modification. Polym J. 2008;40(7):577–589. doi: 10.1295/polymj.PJ2008039

[24] Wulf V, Bisker G. Integrating single-walled carbon nanotubes into supramolecular assemblies: from basic interactions to emerging applications. ACS Nano. 2024;18(43):29380–29393. doi: 10.1021/acsnano.4c06843 39428637 PMC11526426

[25] Ishibashi A, Nakashima N. Strong chemical structure dependence for individual dissolution of single-walled carbon nanotubes in aqueous micelles of biosurfactants. Bull Chem Soc Jpn. 2006;79(2):357–359. doi: 10.1246/bcsj.79.357

[26] Ke PC. Fiddling the string of carbon nanotubes with amphiphiles. Phys Chem Phys. 2007;9(4):439–447. doi: 10.1039/B611142D 17216058

[27] Noguchi Y, Fujigaya T, Niidome Y, et al. Single-walled carbon nanotubes/DNA hybrids in water are highly stable [Article]. Chem Phys Lett. 2008;455(4–6):249–251. doi: 10.1016/j.cplett.2008.02.089

[28] Nakashima N, Okuzono S, Murakami H, et al. Dna dissolves single-walled carbon nanotubes in water. Chem Lett. 2003;32(5):456–457. doi: 10.1246/cl.2003.456

[29] Curran SA, Ajayan PM, Blau WJ, et al. A composite from poly(m-phenylenevinylene-co-2,5-dioctoxy-p-phenylenevinylene) and carbon nanotubes. A novel material for molecular optoelectronics. Adv Mater (Weinheim, Germany). 1998;10(14):1091–1093. doi: 10.1002/(SICI)1521-4095(199810)10:14<1091::AID-ADMA1091>3.0.CO;2-L

[30] Coleman JN, Curran S, Dalton AB, et al. Percolation-dominated conductivity in a conjugated-polymer-carbon-nanotube composite. Phys Rev B. 1998;58(12):R7492–R7495. doi: 10.1103/PhysRevB.58.R7492

[31] Nish A, Hwang J-Y, Doig J, et al. Highly selective dispersion of single-walled carbon nanotubes using aromatic polymers. Nat Nanotechnol. 2007;2(10):640–646. doi: 10.1038/nnano.2007.290 18654390

[32] Fujigaya T, Nakashima N. Fuel cell electrocatalyst using polybenzimidazole-modified carbon nanotubes as support materials. Adv Mater. 2013;25(12):1666–1681. doi: 10.1002/adma.201204461 23423836

[33] Wu J-F, Lo C-F, Li L-Y, et al. Thermally stable polybenzimidazole/carbon nano-tube composites for alkaline direct methanol fuel cell applications. J Power Sources. 2014;246:39–48. doi: 10.1016/j.jpowsour.2013.05.171

[34] Kang JY, Eo SM, Jeon IY, et al. Multifunctional poly(2,5-benzimidazole)/carbon nanotube composite films. J Polym Sci A Polym Chem. 2010;48(5):1067–1078. doi: 10.1002/pola.23862

[35] Lu Y, Chen J, Cui H, et al. In situ synthesis of polybenzimidazole on the surface of single wall carbon nanotubes. J Dispersion Sci Technol. 2009;30(7):1091–1094. doi: 10.1080/01932690802598440

[36] Kannan R, Aher PP, Palaniselvam T, et al. Artificially designed membranes using phosphonated multiwall carbon nanotube−polybenzimidazole composites for polymer electrolyte fuel cells. J Phys Chem Lett. 2010;1(14):2109–2113. doi: 10.1021/jz1007005

[37] Hua M-Y, Chen H-C, Tsai R-Y, et al. Preparation of polybenzimidazole-carboxylated multiwalled carbon nanotube composite for intrinsic sensing of hydrogen peroxide. J Phys Chem C. 2011;115(31):15182–15190. doi: 10.1021/jp202262e

[38] Kannan R, Kagalwala HN, Chaudhari HD, et al. Improved performance of phosphonated carbon nanotube-polybenzimidazole composite membranes in proton exchange membrane fuel cells. J Mater Chem. 2011;21(20):7223–7231. doi: 10.1039/c0jm04265j

[39] Zhang L, Ni Q-Q, Shiga A, et al. Preparation of polybenzimidazole/functionalized carbon nanotube nanocomposite films for use as protective coatings. Polym Eng Sci. 2011;51(8):1525–1532. doi: 10.1002/pen.21618

[40] Okamoto M, Fujigaya T, Nakashima N. Individual dissolution of single-walled carbon nanotubes by using polybenzimidazole, and highly effective reinforcement of their composite films. Adv Funct Mater. 2008;18(12):1776–1782. doi: 10.1002/adfm.200701257

[41] Yoon SH, Park YJ. Polyimide-coated carbon electrodes combined with redox mediators for superior Li-O(2) cells with excellent cycling performance and decreased overpotential. Sci Rep. 2017;7(1):42617. doi: 10.1038/srep42617 28198419 PMC5309741

[42] Ning W, Wang Z, Liu P, et al. Multifunctional super-aligned carbon nanotube/polyimide composite film heaters and actuators. Carbon. 2018;139:1136–1143. doi: 10.1016/j.carbon.2018.08.011

[43] Fujigaya T, Morimoto T, Niidome Y, et al. NIR laser-driven reversible volume phase transition of single-walled carbon nanotube/poly(N-isopropylacrylamide) composite gels [Article]. Adv Mater. 2008;20(19):3610–3614. doi: 10.1002/adma.200800494

[44] Shar JA, Cosgrove T, Obey TM, et al. Adsorption studies of diblock copolymers at the cyclohexane/carbon Black interface. Langmuir. 1999;15(22):7688–7694. doi: 10.1021/la990050l

[45] Modi S, Panwar A, Mead JL, et al. Effect of molecular weight on the electrophoretic deposition of carbon Black nanoparticles in moderately viscous systems. Langmuir. 2013;29(31):9702–9711. doi: 10.1021/la401657d 23848316

[46] Lin Y, Smith TW, Alexandridis P. Adsorption of a rake-type siloxane surfactant onto carbon Black nanoparticles dispersed in aqueous media. Langmuir. 2002;18(16):6147–6158. doi: 10.1021/la011671t

[47] Shibayama M, Matsunaga T, Kusano T, et al. Sans studies on catalyst ink of fuel cell. J Appl Polym Sci. 2014;131(3). doi: 10.1002/app.39842

[48] Nazmul Islam ABM, Kayo N, Motoishi Y, et al. Kinetics and thermodynamics analysis of the polybenzimidazole adsorption onto carbon materials using adsorption isotherm measurements [Article]. Polym J. 2024;56(12):1153–1163. doi: 10.1038/s41428-024-00950-5

[49] Han Z, Motoishi Y, Fujigaya T. Alkaline stability of anion-conductive ionomer coated on a carbon surface. ACS Omega. 2019;4(17):17134–17139. doi: 10.1021/acsomega.9b01466 31656886 PMC6811845

[50] Hafez IH, Berber MR, Fujigaya T, et al. Enhancement of platinum mass activity on the surface of polymer-wrapped carbon nanotube-based fuel cell electrocatalysts [Article]. Sci Rep. 2014;4(1):6295. doi: 10.1038/srep06295 25221915 PMC4164040

[51] Liu X, Huang J, Du Y, et al. Enhanced thermoelectric properties of flexible self-supporting carbon nanotube film/polypyrrole composites. Cell Rep Phys Sci. 2024;5(9):102163. doi: 10.1016/j.xcrp.2024.102163

[52] Almasoudi M, Salah N, Alshahrie A, et al. High thermoelectric power generation by SWCNT/PPy core shell nanocomposites. Nanomaterials (Basel). 2022;12(15):2582. doi: 10.3390/nano12152582 35957013 PMC9370189

[53] Oueiny C, Berlioz S, Perrin F-X. Carbon nanotube–polyaniline composites. Prog Polym Sci. 2014;39(4):707–748. doi: 10.1016/j.progpolymsci.2013.08.009

[54] Peng C, Zhang S, Jewell D, et al. Carbon nanotube and conducting polymer composites for supercapacitors. Prog Nat Sci. 2008;18(7):777–788. doi: 10.1016/j.pnsc.2008.03.002

[55] Schroeder V, Savagatrup S, He M, et al. Carbon nanotube chemical sensors. Chem Rev. 2019;119(1):599–663. doi: 10.1021/acs.chemrev.8b00340 30226055 PMC6399066

[56] Wang Y, Yeow JTW, Penza M. A review of carbon nanotubes‐based gas sensors. J Sensors. 2009;2009(1):1–24. doi: 10.1155/2009/493904

[57] Kauffman DR, Star A. Carbon nanotube gas and vapor sensors. Angew Chem Int Ed Engl. 2008;47(35):6550–6570. doi: 10.1002/anie.200704488 18642264

[58] Meyyappan M. Carbon nanotube-based chemical sensors. Small. 2016;12(16):2118–2129. doi: 10.1002/smll.201502555 26959284

[59] Ellis JE, Star A. Carbon nanotube based gas sensors toward breath analysis. Chempluschem. 2016;81(12):1248–1265. doi: 10.1002/cplu.201600478 31964066

[60] Luo K, Peng H, Zhang B, et al. Advances in carbon nanotube-based gas sensors: exploring the path to the future. Coord Chem Rev. 2024;518:1–40. doi: 10.1016/j.ccr.2024.216049

[61] Guo SY, Hou PX, Zhang F, et al. Gas sensors based on single-wall carbon nanotubes. Molecules. 2022;27(17):5381. doi: 10.3390/molecules27175381 36080149 PMC9458085

[62] Choi SJ, Yoon B, Ray JD, et al. Chemiresistors for the real‐time wireless detection of anions. Adv Funct Mater. 2019;30(7). doi: 10.1002/adfm.201907087

[63] Li B, Sui N, Li M, et al. High-sensitivity and energy-efficient chloride ion sensors based on flexible printed carbon nanotube thin-film transistors for wearable electronics. Talanta. 2024;276:126285. doi: 10.1016/j.talanta.2024.126285 38781918

[64] Yan L, Zhang Y, Zhu Z, et al. Robust carbon nanotube transistor ion sensors with near-nernstian sensitivity for multi-ion detection in neurological diseases. Nanomaterials (basel). 2025;15(6):447. doi: 10.3390/nano15060447 40137620 PMC11945060

[65] Choi SJ, Yoon B, Lin S, et al. Functional single-walled carbon nanotubes for anion sensing. ACS Appl Mater Interface. 2020;12(25):28375–28382. doi: 10.1021/acsami.0c03813 32519847

[66] Fennell JF, Liu SF, Azzarelli JM, et al. Nanowire chemical/biological sensors: status and a roadmap for the future. Angew Chem Int Ed Engl. 2016;55(4):1266–1281. doi: 10.1002/anie.201505308 26661299

[67] Yang N, Chen X, Ren T, et al. Carbon nanotube based biosensors. Sens Actuators B. 2015;207:690–715. doi: 10.1016/j.snb.2014.10.040

[68] Mousavi SM, Nezhad FF, Ghahramani Y, et al. Recent advances in bioactive carbon nanotubes based on polymer composites for biosensor applications. Chem Biodivers. 2024;21(7):e202301288. doi: 10.1002/cbdv.202301288 38697942

[69] Wang J. Carbon‐nanotube based electrochemical biosensors: a review. Electroanalysis. 2005;17(1):7–14. doi: 10.1002/elan.200403113

[70] Saguin NSG, Maulik G, Cao X, et al. Cnts-based biosensors for enzyme detection. Sens Actuators A. 2024;377:115753. doi: 10.1016/j.sna.2024.115753

[71] Ranjbari S, Bolourinezhad M, Kesharwani P, et al. Applications of carbon nanotube biosensors: sensing the future. J Drug Deliv Sci Technol. 2024;97:105747. doi: 10.1016/j.jddst.2024.105747

[72] Meskher H, Mustansar HC, Thakur AK, et al. Recent trends in carbon nanotube (CNT)-based biosensors for the fast and sensitive detection of human viruses: a critical review. Nanoscale Adv. 2023;5(4):992–1010. doi: 10.1039/D2NA00236A 36798507 PMC9926911

[73] Dewey HM, Lamb A, Budhathoki-Uprety J. Recent advances on applications of single-walled carbon nanotubes as cutting-edge optical nanosensors for biosensing technologies. Nanoscale. 2024;16(35):16344–16375. doi: 10.1039/D4NR01892C 39157856

[74] Feng B, Zhao W, Zhang M, et al. Lignin-based carbon nanomaterials for biochemical sensing applications. Chem Asian J. 2024;19(19):e202400611. doi: 10.1002/asia.202400611 38995858

[75] Kim I-D, Rothschild A, Tuller HL. Advances and new directions in gas-sensing devices. Acta Mater. 2013;61(3):974–1000. doi: 10.1016/j.actamat.2012.10.041

[76] Zhou X, Lee S, Xu Z, et al. Recent progress on the development of chemosensors for gases. Chem Rev. 2015;115(15):7944–8000. doi: 10.1021/cr500567r 25651137

[77] Jung S, Roman C, Hierold C. Fast nitrogen dioxide sensing with ultralow‐power nanotube gas sensors. Adv Sens Res. 2023;3(1). doi: 10.1002/adsr.202300081

[78] Zuo H, Zhan S, Xu W, et al. Theoretical approaches toward designing sensitive materials for carbon nanotube-based field-effect transistor gas sensors. Sens Actuators B. 2024;409:409. doi: 10.1016/j.snb.2024.135604

[79] Jing Kong NRF, Zhou C, Chapline MG, et al. Nanotube molecular wires as chemical sensors. Science. 2000;287(5453):287. doi: 10.1126/science.287.5453.622 10649989

[80] Shiv Dutta Lawaniya SK, Yeontae Y, Horst-Günter R, et al. Functional nanomaterials in flexible gas sensors recent progress and future prospects. Mater Today Chem. 2023;29(101428):1.

[81] Kurugundla Gopi Krishna SP, Pothukanuri N, Kathirvelu V, et al. Nanostructured metal oxide semiconductor-based gas sensors a comprehensive review. Sens Actuators. 2022;341(113578):1.

[82] Battie Y, Ducloux O, Thobois P, et al. Gas sensors based on thick films of semi-conducting single walled carbon nanotubes. Carbon. 2011;49(11):3544–3552. doi: 10.1016/j.carbon.2011.04.054

[83] Hong HP, Kim JH, Lee CJ, et al. In-plane impedancemetric ammonia sensing of solution-deposited, highly semiconductor-enriched single-wall carbon nanotube submonolayer network gas sensors. Sens Actuators B. 2015;220:27–32. doi: 10.1016/j.snb.2015.05.014

[84] Tian T, Yin H, Zhang L, et al. Gas sensing performance and charge-transfer mechanism of semiconducting single-walled carbon nanotubes. Appl Surf Sci. 2023;609:609. doi: 10.1016/j.apsusc.2022.155357

[85] Yang H, Neal L, Flores EE, et al. Role and impact of surfactants in carbon nanotube dispersions and sorting. J Surfact Deterg. 2023;26(5):607–622. doi: 10.1002/jsde.12702

[86] Wei X, Li S, Wang W, et al. Recent advances in structure separation of single-wall carbon nanotubes and their application in optics, electronics, and optoelectronics. Adv Sci (Weinh). 2022;9(14):e2200054. doi: 10.1002/advs.202200054 35293698 PMC9108629

[87] Wang J, Lei T. Separation of semiconducting carbon nanotubes using conjugated polymer wrapping. Polymers (Basel). 2020;12(7):1548. doi: 10.3390/polym12071548 32668780 PMC7407812

[88] Liang L, Xie W, Fang S, et al. High-efficiency dispersion and sorting of single-walled carbon nanotubes via non-covalent interactions. J Mater Chem C. 2017;5(44):11339–11368. doi: 10.1039/C7TC04390B

[89] Wang H, Bao Z. Conjugated polymer sorting of semiconducting carbon nanotubes and their electronic applications. Nano Today. 2015;10(6):737–758. doi: 10.1016/j.nantod.2015.11.008

[90] Du H, Maimaitiyiming X, Luo Y, et al. A highly sensitive ammonia gas sensor based on non-covalent functionalized single-walled carbon nanotubes with Schiff base polyphenylene polymer. Sens Actuators B. 2023;394:134426. doi: 10.1016/j.snb.2023.134426

[91] Zhang J, Maimaitiyiming X, Obolda A. Differential detection of NH3 and NO2 by polymer non-covalently encapsulated semiconducting single-walled carbon nanotubes. Sens Actuators B. 2024;418:418. doi: 10.1016/j.snb.2024.136199

[92] Nie P, Zhang J, Du H, et al. Double-bounded diphenyl-contained fluorene polymers for Sc-SWCNTs composite to ammonia gas sensors: diphenylethene or diphenylmethanimine. Dyes Pigm. 2023;220:220. doi: 10.1016/j.dyepig.2023.111690

[93] Nie P, Wu H, Maimaitiyiming X. Polycarbazole-based polymer/semiconductor carbon nanotube composites for NO2 and NH3 gas sensing applications. Sep Purif Technol. 2025;363:132038. doi: 10.1016/j.seppur.2025.132038

[94] Zhang J, Maimaitiyiming X. Study on phenyl series of polymers wrapping of different diameters of semiconducting single-walled carbon nanotubes for ammonia gas sensors. Sens Actuators B. 2024;405:405. doi: 10.1016/j.snb.2024.135278

[95] Zhang W, Liu H, Sun C, et al. Capturing CO2 from ambient air using a polyethyleneimine–silica adsorbent in fluidized beds. Chem Eng Sci. 2014;116:306–316. doi: 10.1016/j.ces.2014.05.018

[96] Heydari-Gorji A, Sayari A. Co2 capture on polyethylenimine-impregnated hydrophobic mesoporous silica: experimental and kinetic modeling. Chem Eng J. 2011;173(1):72–79. doi: 10.1016/j.cej.2011.07.038

[97] Wang J, Chen H, Zhou H, et al. Carbon dioxide capture using polyethylenimine-loaded mesoporous carbons. J Environ Sci (China). 2013;25(1):124–132. doi: 10.1016/S1001-0742(12)60011-4 23586307

[98] Li K, Jiang J, Yan F, et al. The influence of polyethyleneimine type and molecular weight on the CO2 capture performance of PEI-nano silica adsorbents. Appl Energy. 2014;136:750–755. doi: 10.1016/j.apenergy.2014.09.057

[99] Choi W, Min K, Kim C, et al. Epoxide-functionalization of polyethyleneimine for synthesis of stable carbon dioxide adsorbent in temperature swing adsorption. Nat Commun. 2016;7(1):12640. doi: 10.1038/ncomms12640 27572662 PMC5013602

[100] Siefker ZA, Boyina A, Braun JE, et al. A chemiresistive CO2 sensor based on CNT-functional polymer composite films. 2020 IEEE Sensors. 2020:1–4.

[101] Manzoor S, Talib M, Arsenin AV, et al. Polyethyleneimine-starch functionalization of single-walled carbon nanotubes for carbon dioxide sensing at room temperature. ACS Omega. 2023;8(1):893–906. doi: 10.1021/acsomega.2c06243 36643491 PMC9835164

[102] Bezdek MJ, Luo SL, Ku KH, et al. A chemiresistive methane sensor. Proc Natl Acad Sci USA. 2021;118(2):e2022515118. 33384329 10.1073/pnas.2022515118 PMC7812817

[103] Dunlap JH, Feng H, Pioch T, et al. Continuous flow chemistry and Bayesian optimization for polymer-functionalized carbon nanotube-based chemiresistive methane sensors. ACS Appl Mater Interface. 2024;16(49):68181–68196. doi: 10.1021/acsami.4c14279 PMC11647762 39592136

[104] Luo SL, Yuan W, Xue M, et al. Chemiresistive hydrogen sensing with size-limited palladium nanoparticles in iptycene-containing poly(arylene ether)s. ACS Nano. 2023;17(3):2679–2688. doi: 10.1021/acsnano.2c10736 36639134 PMC12145736

[105] Tang X, Girma HG, Li Z, et al. “Dragging mode” electrohydrodynamic jet printing of polymer-wrapped semiconducting single-walled carbon nanotubes for no gas-sensing field-effect transistors. J Mater Chem C. 2021;9(44):15804–15812. doi: 10.1039/D1TC04638A

[106] Guo C, Ouyang J, Shin H, et al. Enrichment of semiconducting single-walled carbon nanotubes with indigo-fluorene-based copolymers and their use in printed thin-film transistors and carbon dioxide gas sensors. ACS Sens. 2020;5(7):2136–2145. doi: 10.1021/acssensors.0c00764 32519539

[107] Girma HG, Park KH, Ji D, et al. Room‐temperature hydrogen sensor with high sensitivity and selectivity using chemically immobilized monolayer single‐walled carbon nanotubes. Adv Funct Mater. 2023;33(18). doi: 10.1002/adfm.202213381

[108] Zhu Z, Liu H, Luo X, et al. Ultrasensitive ion liquid-CNT vdW heterostructure interface via anion-cation pair switch for SO2 detection. Sens Actuators B. 2024;13(10):412. doi: 10.3390/act13100412

[109] Li C, Chen S, Liu C, et al. A flexible multi-ion detection system based on organic electrochemical transistors for physiological monitoring. Electronics. 2025;14(5):1023. doi: 10.3390/electronics14051023

[110] Gil M, Rudy M, Duma-Kocan P, et al. Electronic sensing technologies in food quality assessment: a comprehensive literature review. Appl Sci. 2025;15(3):1530. doi: 10.3390/app15031530

[111] Bucella SG, Salazar‐Rios JM, Derenskyi V, et al. Inkjet printed single‐walled carbon nanotube based ambipolar and unipolar transistors for high‐performance complementary logic circuits. Adv Elect Mater. 2016;2(6). doi: 10.1002/aelm.201600094

[112] Kim B, Geier ML, Hersam MC, et al. Inkjet printed circuits based on ambipolar and p-type carbon nanotube thin-film transistors. Sci Rep. 2017;7(1):39627. doi: 10.1038/srep39627 28145438 PMC5286420

[113] Rother M, Brohmann M, Yang S, et al. Aerosol‐jet printing of polymer‐sorted (6,5) carbon nanotubes for field‐effect transistors with high reproducibility. Adv Elect Mater. 2017;3(8). doi: 10.1002/aelm.201700080

[114] Cao C, Andrews JB, Franklin AD. Completely printed, flexible, stable, and hysteresis‐free carbon nanotube thin‐film transistors via aerosol jet printing. Adv Elect Mater. 2017;3(5). doi: 10.1002/aelm.201700057

[115] Ha M, Seo JW, Prabhumirashi PL, et al. Aerosol jet printed, low voltage, electrolyte gated carbon nanotube ring oscillators with sub-5 μs stage delays. Nano Lett. 2013;13(3):954–960. 23394463 10.1021/nl3038773

[116] Schneider S, Lefebvre J, Diercks NJ, et al. Phenanthroline additives for enhanced semiconducting carbon nanotube dispersion stability and transistor performance. ACS Appl Nano Mater. 2020;3(12):12314–12324. doi: 10.1021/acsanm.0c02813

[117] Liu L, Jh LX, Zhou C, et al. Aligned, high-density semiconducting carbon nanotube arrays for high-performance electronics. Science. 2020;368(6493):850–856. doi: 10.1126/science.aba5980 32439787

[118] Gomulya W, Gao J, Loi MA. Conjugated polymer-wrapped carbon nanotubes: physical properties and device applications. Eur Phys J B. 2013;86(10). doi: 10.1140/epjb/e2013-40707-9

[119] Ozawa H, Ide N, Fujigaya T, et al. One-pot separation of highly enriched (6,5)-single-walled carbon nanotubes using a fluorene-based copolymer [Article]. Chem Lett. 2011;40(3):239–241. doi: 10.1246/cl.2011.239

[120] Balci Leinen M, Lindenthal S, Heimfarth D, et al. Networks of as-dispersed, polymer-wrapped (6,5) single-walled carbon nanotubes for selective Cu(2+) and glyphosate sensing. Nanoscale. 2022;14(37):13542–13550. doi: 10.1039/D2NR02517E 36097951

[121] Heimfarth D, Balci Leinen M, Klein P, et al. Enhancing electrochemical transistors based on polymer-wrapped (6,5) carbon nanotube networks with ethylene glycol side chains. ACS Appl Mater Interface. 2022;14(6):8209–8217. doi: 10.1021/acsami.1c23586 35108486

[122] Zhang J, Landry MP, Barone PW, et al. Molecular recognition using corona phase complexes made of synthetic polymers adsorbed on carbon nanotubes. Nat Nanotechnol. 2013;8(12):959–968. doi: 10.1038/nnano.2013.236 24270641 PMC5051352

[123] Lew TTS, Koman VB, Gordiichuk P, et al. The emergence of plant nanobionics and living plants as technology. Adv Mater Technol. 2019;5(3):1900657.

[124] Khong DT, Tan GZH, Cheerlavancha R, et al. Nanosensor for Fe(II) and Fe(III) allowing spatiotemporal sensing in planta. Nano Lett. 2025;25(6):2316–2324. doi: 10.1021/acs.nanolett.4c05600 39873722

[125] Park M, Salem DP, Parviz D, et al. Measuring the accessible surface area within the nanoparticle corona using molecular probe adsorption. Nano Lett. 2019;19(11):7712–7724. doi: 10.1021/acs.nanolett.9b02647 31565943 PMC7206615

[126] Blackburn JL, Ferguson AJ, Cho C, et al. Carbon‐nanotube‐based thermoelectric materials and devices. Adv Mater. 2018;30(11). doi: 10.1002/adma.201704386 29356158

[127] Zhou S, Shi XL, Li L, et al. Advances and outlooks for carbon nanotube-based thermoelectric materials and devices. Adv Mater. 2025;37(13):e2500947. doi: 10.1002/adma.202500947 39955649 PMC11962713

[128] Hicks LD, Dresselhaus MS. Thermoelectric figure of merit of a one-dimensional conductor [Article]. Phys Rev B. 1993;47(24):16631–16634. doi: 10.1103/PhysRevB.47.16631 10006109

[129] Yang J, Zhang H, Hu N, et al. Recent advances in carbon nanotubes-based organic thermoelectric composites-a mini review. Mater Today Nano. 2025;29:100590. doi: 10.1016/j.mtnano.2025.100590

[130] Hu X, Bao X, Zhang M, et al. Recent advances in carbon nanotube-based energy harvesting technologies. Adv Mater. 2023;35(49):e2303035. doi: 10.1002/adma.202303035 37209369

[131] Liu Y, Zhao Z, Kang L, et al. Molecular doping modulation and applications of structure-sorted single-walled carbon nanotubes: a review. Small. 2024;20(3):e2304075. doi: 10.1002/smll.202304075 37675833

[132] Zhang S, Pang J, Li Y, et al. Emerging Internet of Things driven carbon nanotubes-based devices. Nano Res. 2022;15(5):4613–4637. doi: 10.1007/s12274-021-3986-7

[133] Hong CT, Lee W, Kang YH, et al. Effective doping by spin-coating and enhanced thermoelectric power factors in SWCNT/P3HT hybrid films. J Mater Chem A. 2015;3(23):12314–12319. doi: 10.1039/C5TA02443A

[134] Lee W, Hong CT, Kwon OH, et al. Enhanced thermoelectric performance of bar-coated SWCNT/P3HT thin films. ACS Appl Mater Interface. 2015;7(12):6550–6556. doi: 10.1021/acsami.5b00626 25762308

[135] Hong CT, Kang YH, Ryu J, et al. Spray-printed CNT/P3HT organic thermoelectric films and power generators. J Mater Chem A. 2015;3(43):21428–21433. doi: 10.1039/C5TA06096F

[136] Lee W, Kang YH, Lee JY, et al. Hot-pressing for improving performance of CNT/conjugated polymer thermoelectric films and power generators. Mater Today Commun. 2017;10:41–45. doi: 10.1016/j.mtcomm.2016.12.002

[137] He P, Shimano S, Salikolimi K, et al. Noncovalent modification of single-walled carbon nanotubes using thermally cleavable polythiophenes for solution-processed thermoelectric films. ACS Appl Mater Interface. 2019;11(4):4211–4218. doi: 10.1021/acsami.8b14820 30516052

[138] Hong S-H, Lee T-C, Liu C-L. All-solution-processed polythiophene/carbon nanotube nanocomposites integrated on biocompatible silk fibroin substrates for wearable thermoelectric generators. ACS Appl Energy Mater. 2023;6(4):2602–2610. doi: 10.1021/acsaem.2c04160

[139] Chen X, Chen S, Wang D, et al. The influence of molecular weights on dispersion and thermoelectric performance of alkoxy side-chain polythiophene/carbon nanotube composite materials. Polymers (Basel). 2024;16(17):2444. doi: 10.3390/polym16172444 39274077 PMC11397576

[140] Nishiyama K, Hsiao Y-T, Wu W-N, et al. Controlled synthesis of alkylthio-substituted poly(thienylene vinylene) and its carbon nanotube composites for enhanced thermoelectric performance. J Mater Chem A. 2025;13(5):3551–3561. doi: 10.1039/D4TA07611G

[141] Zhang Y, Yang Q, Lin C, et al. Enhanced thermoelectric and mechanical performance of fully conjugated block polythiophene modified with polar ethylene glycol side chains/single-walled carbon nanotube composite materials. J Mater Chem A. 2024;12(42):29262–29270. doi: 10.1039/D4TA05540C

[142] Lin P-S, Inagaki S, Liu J-H, et al. The role of branched alkylthio side chain on dispersion and thermoelectric properties of regioregular polythiophene/carbon nanotubes nanocomposites. Chem Eng J. 2023;458:458. doi: 10.1016/j.cej.2023.141366

[143] Hao L, Kang J, Shi J, et al. Enhanced thermoelectric performance of poly(3-substituted thiophene)/single-walled carbon nanotube composites via polar side chain modification. Compos Sci Technol. 2020;199:199. doi: 10.1016/j.compscitech.2020.108359

[144] Liu C, Yin X, Chen Z, et al. Improving the thermoelectric performance of solution-processed polymer nanocomposites by introducing platinum acetylides with tailored intermolecular interactions. Chem Eng J. 2021;419:419. doi: 10.1016/j.cej.2021.129624

[145] Li X, Zhu Z, Wang T, et al. Improved thermoelectric performance of P3HT/SWCNTs composite films by HClO4 post-treatment. Compos Commun. 2019;12:128–132. doi: 10.1016/j.coco.2019.01.009

[146] Finn PA, Jacobs IE, Armitage J, et al. Effect of polar side chains on neutral and p-doped polythiophene. J Mater Chem C. 2020;8(45):16216–16223. doi: 10.1039/D0TC04290K

[147] Zheng Q-B, Lin Y-C, Lin Y-T, et al. Investigating the stretchability of doped poly(3-hexylthiophene)-block-poly(butyl acrylate) conjugated block copolymer thermoelectric thin films. Chem Eng J. 2023;472:472. doi: 10.1016/j.cej.2023.145121

[148] Cp G, B K, I M, et al. Extreme oxygen sensitivity of electronic properties of carbon nanotubes. Science. 2000;287(5459):287(5459). doi: 10.1126/science.287.5459.1801 10710305

[149] Ryu Y, Freeman D, Yu C. High electrical conductivity and n-type thermopower from double-/single-wall carbon nanotubes by manipulating charge interactions between nanotubes and organic/inorganic nanomaterials. Carbon. 2011;49(14):4745–4751. doi: 10.1016/j.carbon.2011.06.082

[150] Piao M, Alam MR, Kim G, et al. Effect of chemical treatment on the thermoelectric properties of single walled carbon nanotube networks. Physica Status Solidi (B). 2012;249(12):2353–2356. doi: 10.1002/pssb.201200101

[151] Nonoguchi Y, Ohashi K, Kanazawa R, et al. Systematic conversion of single walled carbon nanotubes into n-type thermoelectric materials by molecular dopants. Sci Rep. 2013;3(1):3344. doi: 10.1038/srep03344 24276090 PMC3840380

[152] Horike S, Fukushima T, Saito T, et al. Highly stable n-type thermoelectric materials fabricated via electron doping into inkjet-printed carbon nanotubes using oxygen-abundant simple polymers. Mol Syst Des Eng. 2017;2(5):616–623. doi: 10.1039/C7ME00063D

[153] Zhang L, Shang H, Zou Q, et al. High-power-density and excellent-flexibility thermoelectric generator based on all-SWCNTs/PVP composites. Small. 2024;20(27):e2306125. doi: 10.1002/smll.202306125 38282085

[154] Zhou W, Fan Q, Zhang Q, et al. High-performance and compact-designed flexible thermoelectric modules enabled by a reticulate carbon nanotube architecture. Nat Commun. 2017;8(1):14886. doi: 10.1038/ncomms14886 28337987 PMC5477522

[155] Wang Y, Dai X, Pan J, et al. Solvent effect induced charge polarity switching from p- to n-type in polyaniline and carbon nanotube hybrid films with a high thermoelectric power factor. J Mater Chem A. 2024;12(30):18948–18957. doi: 10.1039/D4TA02790F

[156] Yao Q, Wang Q, Wang L, et al. Abnormally enhanced thermoelectric transport properties of SWNT/PANI hybrid films by the strengthened PANI molecular ordering. Energy Environ Sci. 2014;7(11):3801–3807. doi: 10.1039/C4EE01905A

[157] Chen J, Wang L, Gui X, et al. Strong anisotropy in thermoelectric properties of CNT/PANI composites. Carbon. 2017;114:1–7. doi: 10.1016/j.carbon.2016.11.074

[158] Li H, Liang Y, Liu Y, et al. Engineering doping level for enhanced thermoelectric performance of carbon nanotubes/polyaniline composites. Compos Sci Technol. 2021;210:108797. doi: 10.1016/j.compscitech.2021.108797

[159] Yin S, Lu W, Wu X, et al. Enhancing thermoelectric performance of polyaniline/single-walled carbon nanotube composites via dimethyl sulfoxide-mediated electropolymerization. ACS Appl Mater Interface. 2021;13(3):3930–3936. doi: 10.1021/acsami.0c19100 33455158

[160] Wang L, Yao Q, Xiao J, et al. Engineered molecular chain ordering in single-walled carbon nanotubes/polyaniline composite films for high-performance organic thermoelectric materials. Chem Asian J. 2016;11(12):1804–1810. doi: 10.1002/asia.201600212 27123885

[161] Yu P, Wu R, Liu C, et al. Polyaniline/SWCNT composite films prepared via the solvent-induced strategy for flexible energy harvesting. Sustain Energy Fuels. 2023;7(1):172–180. doi: 10.1039/D2SE01295B

[162] Li H, Liu Y, Liu S, et al. Wet-spun flexible carbon nanotubes/polyaniline fibers for wearable thermoelectric energy harvesting. Compos Part A: Appl Sci Manuf. 2023;166:166. doi: 10.1016/j.compositesa.2022.107386

[163] Feng L, Wu R, Liu C, et al. Facile green vacuum-assisted method for polyaniline/SWCNT hybrid films with enhanced thermoelectric performance by interfacial morphology control. ACS Appl Energy Mater. 2021;4(4):4081–4089. doi: 10.1021/acsaem.1c00419

[164] Wang S, Liu F, Gao C, et al. Enhancement of the thermoelectric property of nanostructured polyaniline/carbon nanotube composites by introducing pyrrole unit onto polyaniline backbone via a sustainable method. Chem Eng J. 2019;370:322–329. doi: 10.1016/j.cej.2019.03.155

[165] Wang H, S-I Y, Yu C. Engineering electrical transport at the interface of conjugated carbon structures to improve thermoelectric properties of their composites. Polymer. 2016;97:487–495.

[166] Qin Yao LC, Zhang W, Liufu S, et al. Enhanced thermoelectric performance of single-walled carbon nanotubes/polyaniline hybrid nanocomposites. ACS Nano. 2010;4(4):2445–2451. doi: 10.1021/nn1002562 20359234

[167] Liu J, Sun J, Gao L. Flexible single-walled carbon nanotubes/polyaniline composite films and their enhanced thermoelectric properties. Nanoscale. 2011;3(9):3616–3619. doi: 10.1039/c1nr10386e 21826327

[168] Wu R, Yuan H, Liu C, et al. Flexible PANI/SWCNT thermoelectric films with ultrahigh electrical conductivity. RSC Adv. 2018;8(46):26011–26019. doi: 10.1039/C8RA04863K 35541936 PMC9082852

[169] Mistry KS, Larsen BA, Bergeson JD, et al. N-type transparent conducting films of small molecule and polymer amine doped single-walled carbon nanotubes. ACS Nano. 2011;5(5):3714–3723. doi: 10.1021/nn200076r 21388221

[170] Yu C, Murali A, Choi K, et al. Air-stable fabric thermoelectric modules made of n- and p-type carbon nanotubes. Energy Environ Sci. 2012;5(11):9481–9486. doi: 10.1039/c2ee22838f

[171] Nonoguchi Y, Nakano M, Murayama T, et al. Simple salt-coordinated n-type nanocarbon materials stable in air. Adv Funct Mater. 2016;26(18):3021–3028. doi: 10.1002/adfm.201600179

[172] Nakashima Y, Yamaguchi R, Toshimitsu F, et al. Air-stable n-type single-walled carbon nanotubes doped with benzimidazole derivatives for thermoelectric conversion and their air-stable mechanism [Article]. ACS Appl Nano Mater. 2019;2(8):4703–4710. doi: 10.1021/acsanm.9b01174

[173] Erden F, Li H, Wang X, et al. High-performance thermoelectric materials based on ternary TiO(2)/CNT/PANI composites. Phys Chem Chem Phys. 2018;20(14):9411–9418. 29565069 10.1039/C7CP07896J

[174] Meng C, Liu C, Fan S. A promising approach to enhanced thermoelectric properties using carbon nanotube networks. Adv Mater. 2010;22(4):535–539. doi: 10.1002/adma.200902221 20217749

[175] Kang YH, Lee U-H, Jung IH, et al. Enhanced thermoelectric performance of conjugated polymer/CNT nanocomposites by modulating the potential barrier difference between conjugated polymer and CNT. ACS Appl Electron Mater. 2019;1(7):1282–1289. doi: 10.1021/acsaelm.9b00224

[176] Jang JG, Kim T-H, Kim SH, et al. Barrier energy engineering enables efficient carrier transport of single-walled carbon nanotube–small organic molecules hybrid thermoelectrics. ACS Appl Mater Interface. 2025;17(1):857–866. doi: 10.1021/acsami.4c13964 39705599

[177] Hsu J-H, Yu C. Sorting-free utilization of semiconducting carbon nanotubes for large thermoelectric responses. Nano Energy. 2020;67:67. doi: 10.1016/j.nanoen.2019.104282

[178] Li D, Luo C, Chen Y, et al. High performance polymer thermoelectric composite achieved by carbon-coated carbon nanotubes network. ACS Appl Energy Mater. 2019;2(4):2427–2434. doi: 10.1021/acsaem.9b00334

[179] Wei S, Zhang Y, Lv H, et al. SWCNT network evolution of PEDOT: PSS/SWCNT composites for thermoelectric application. Chem Eng J. 2022;428:428. doi: 10.1016/j.cej.2021.131137

[180] Li H, Liu Y, Li P, et al. Enhanced thermoelectric performance of carbon nanotubes/polyaniline composites by multiple interface engineering. ACS Appl Mater Interface. 2021;13(5):6650–6658. doi: 10.1021/acsami.0c20931 33517651

[181] Li H, Liang Y, Liu S, et al. Modulating carrier transport for the enhanced thermoelectric performance of carbon nanotubes/polyaniline composites. Org Electron. 2019;69:62–68. doi: 10.1016/j.orgel.2019.03.006

[182] Liu Z, Sun J, Song H, et al. High performance polypyrrole/SWCNTs composite film as a promising organic thermoelectric material. RSC Adv. 2021;11(29):17704–17709. doi: 10.1039/D1RA02733F 35480213 PMC9033191

[183] Gao C, Chen G. A new strategy to construct thermoelectric composites of SWCNTs and poly-Schiff bases with 1,4-diazabuta-1,3-diene structures acting as bidentate-chelating units. J Mater Chem A. 2016;4(29):11299–11306. doi: 10.1039/C6TA03988J

[184] Yanagi K, Kanda S, Oshima Y, et al. Tuning of the thermoelectric properties of one-dimensional material networks by electric double layer techniques using ionic liquids. Nano Lett. 2014;14(11):6437–6442. doi: 10.1021/nl502982f 25302572

[185] Avery AD, Zhou BH, Lee J, et al. Tailored semiconducting carbon nanotube networks with enhanced thermoelectric properties. Nat Energy. 2016;1(4):16033. doi: 10.1038/nenergy.2016.33

[186] MacLeod BA, Stanton NJ, Gould IE, et al. Large n- and p-type thermoelectric power factors from doped semiconducting single-walled carbon nanotube thin films. Energy Environ Sci. 2017;10(10):2168–2179. doi: 10.1039/C7EE01130J

[187] Nakai Y, Honda K, Yanagi K, et al. Giant Seebeck coefficient in semiconducting single-wall carbon nanotube film. Appl Phys Express. 2014;7(2):025103. doi: 10.7567/APEX.7.025103

[188] Komoto J, Goto C, Kawai T, et al. Rational primary structure design for boosting the thermoelectric properties of semiconducting carbon nanotube networks. Appl Phys Lett. 2021;118(26). doi: 10.1063/5.0055640

[189] Huang W, Toshimitsu F, Ozono K, et al. Thermoelectric properties of dispersant-free semiconducting single-walled carbon nanotubes sorted by a flavin extraction method [Article]. Chem Commun. 2019;55(18):2636–2639. doi: 10.1039/C8CC10264C 30742161

[190] Yagi T, Yoshida K, Sakurai S, et al. Semiconducting carbon nanotube extraction enabled by alkylated cellulose wrapping. J Am Chem Soc. 2024;146(30):20913–20918. doi: 10.1021/jacs.4c05468 38934730

[191] Hung NT, Nugraha ART, Hasdeo EH, et al. Diameter dependence of thermoelectric power of semiconducting carbon nanotubes. Phys Rev B. 2015;92(16):165426. doi: 10.1103/PhysRevB.92.165426

[192] Mistry KS, Larsen BA, Blackburn JL. High-yield dispersions of large-diameter semiconducting single-walled carbon nanotubes with tunable narrow chirality distributions. ACS Nano. 2013;7(3):2231–2239. doi: 10.1021/nn305336x 23379962

[193] Ozawa H, Fujigaya T, Niidome Y, et al. Rational concept to recognize/extract single-walled carbon nanotubes with a specific chirality [Article]. J Am Chem Soc. 2011;133(8):2651–2657. doi: 10.1021/ja109399f 21291252

[194] Fukumaru T, Toshimitsu F, Fujigaya T, et al. Effects of the chemical structure of polyfluorene on selective extraction of semiconducting single-walled carbon nanotubes [Article]. Nanoscale. 2014;6(11):5879–5886. doi: 10.1039/c4nr00809j 24752456

[195] Akazaki K, Toshimitsu F, Ozawa H, et al. Recognition and one-pot extraction of right- and left-handed semiconducting single-walled carbon nanotube enantiomers using fluorene-binaphthol chiral copolymers [Article]. J Am Chem Soc. 2012;134(30):12700–12707. doi: 10.1021/ja304244g 22788840

[196] Yi X, Ozawa H, Nakagawa G, et al. Single-walled carbon nanotube thin film transistor fabricated using solution prepared with 9,9-dioctyfluorenyl-2,7-diyl-bipyridine copolymer [Article]. Jpn J Appl Phys. 2011;50(7 PART 1):070207. doi: 10.1143/JJAP.50.070207

[197] Ozawa H, Yi X, Fujigaya T, et al. Supramolecular hybrid of gold nanoparticles and semiconducting single-walled carbon nanotubes wrapped by a porphyrin-fluorene copolymer [Article]. J Am Chem Soc. 2011;133(37):14771–14777. doi: 10.1021/ja2055885 21827201

[198] Ozawa H, Ide N, Fujigaya T, et al. Supramolecular hybrid of metal nanoparticles and semiconducting single-walled carbon nanotubes wrapped by a fluorene-carbazole copolymer [Article]. Chem A Eur J. 2011;17(48):13438–13444. doi: 10.1002/chem.201101669 22068876

[199] Ozawa H, Fujigaya T, Song S, et al. Different chiral selective recognition/extraction of (n,m)single-walled carbon nanotubes using copolymers carrying a carbazole or fluorene moiety [Article]. Chem Lett. 2011;40(5):470–472. doi: 10.1246/cl.2011.470

[200] Ozawa H, Fujigaya T, Niidome Y, et al. Effect of backbone chemical structure of polymers on selective (n,m)single-walled carbon nanotube recognition/extraction behavior [Article]. Chem An Asian J. 2011;6(12):3281–3285. doi: 10.1002/asia.201100362 21936058

[201] Toshimitsu F, Nakashima N. Semiconducting single-walled carbon nanotubes sorting with a removable solubilizer based on dynamic supramolecular coordination chemistry [Article]. Nat Commun. 2014;5(1). doi: 10.1038/ncomms6041 25277810

[202] Graf A, Zakharko Y, Schießl SP, et al. Large scale, selective dispersion of long single-walled carbon nanotubes with high photoluminescence quantum yield by shear force mixing. Carbon. 2016;105:593–599. doi: 10.1016/j.carbon.2016.05.002

[203] Norton-Baker B, Ihly R, Gould IE, et al. Polymer-free carbon nanotube thermoelectrics with improved charge carrier transport and power factor. ACS Energy Lett. 2016;1(6):1212–1220. doi: 10.1021/acsenergylett.6b00417

[204] Lindenthal S, Rippel D, Kistner L, et al. Synergistic p-doping of polymer-wrapped small-diameter single-walled carbon nanotubes by tris(pentafluorophenyl)borane. J Phys Chem C. 2025;129(11):5520–5529. doi: 10.1021/acs.jpcc.4c08584 PMC11931539 40134510

[205] Hawkey A, Dash A, Rodriguez-Martinez X, et al. Ion-exchange doping of semiconducting single-walled carbon nanotubes. Adv Mater. 2024;36(39):e2404554. doi: 10.1002/adma.202404554 39104286

[206] Wang H, Yu C. Organic thermoelectrics: materials preparation, performance optimization, and device integration. Joule. 2019;3(1):53–80. doi: 10.1016/j.joule.2018.10.012

[207] Liang J, Cui R, Zhang X, et al. Polymer/carbon composites with versatile interfacial interactions for high performance carbon‐based thermoelectrics: principles and applications. Adv Funct Mater. 2022;33(9). doi: 10.1002/adfm.202208813

[208] Liu Z, Chen G. Advancing flexible thermoelectric devices with polymer composites. Adv Mater Technol. 2020;5(7). doi: 10.1002/admt.202000049

[209] Spitalsky Z, Tasis D, Papagelis K, et al. Carbon nanotube–polymer composites: chemistry, processing, mechanical and electrical properties. Prog Polym Sci. 2010;35(3):357–401. doi: 10.1016/j.progpolymsci.2009.09.003

[210] Jang JG, Kim TH, Kim SH, et al. Barrier energy engineering enables efficient carrier transport of single-walled carbon nanotube-small organic molecules hybrid thermoelectrics. ACS Appl Mater Interface. 2025;17(1):857–866. 10.1021/acsami.4c13964 39705599

[211] Mai C-K, Liu J, Evans CM, et al. Anisotropic thermal transport in thermoelectric composites of conjugated polyelectrolytes/single-walled carbon nanotubes. Macromolecules. 2016;49(13):4957–4963. doi: 10.1021/acs.macromol.6b00546

[212] Bindl DJ, Shea MJ, Arnold MS. Enhancing extraction of photogenerated excitons from semiconducting carbon nanotube films as photocurrent. Chem Phys. 2013;413 29–34. doi: 10.1016/j.chemphys.2012.08.001

[213] Rdest M, Janas D. Effective doping of single-walled carbon nanotubes with polyethyleneimine. Materials (Basel). 2020;14(1):65. doi: 10.3390/ma14010065 33375643 PMC7795803

[214] MacLeod BA, Stanton NJ, Gould IE, et al. Large n-and p-type thermoelectric power factors from doped semiconducting single-walled carbon nanotube thin films. Energy Environ Sci. 2017;10(10):2168–2179.

[215] Borup R, Meyers J, Pivovar B, et al. Scientific aspects of polymer electrolyte fuel cell durability and degradation. Chem Rev. 2007;107(10):3904–3951. doi: 10.1021/cr050182l 17850115

[216] Okamoto M, Fujigaya T, Nakashima N. Design of an assembly of poly(benzimidazole), carbon nanotubes, and Pt nanoparticles for a fuel-cell electrocatalyst with an ideal interfacial nanostructure. Small. 2009;5(6):735–740. doi: 10.1002/smll.200801742 19263429

[217] Li W, Liang C, Zhou W, et al. Homogeneous and controllable Pt particles deposited on multi-wall carbon nanotubes as cathode catalyst for direct methanol fuel cells. Carbon. 2004;42(2):436–439. doi: 10.1016/j.carbon.2003.10.033

[218] Matsumoto T, Komatsu T, Arai K, et al. Reduction of pt usage in fuel cell electrocatalysts with carbon nanotube electrodes. Chem Commun. 2004;2004(7):840–841. doi: 10.1039/b400607k 15045090

[219] Kongkanand A, Vinodgopal K, Kuwabata S, et al. Highly dispersed Pt catalysts on single-walled carbon nanotubes and their role in methanol oxidation. J Phys Chem B. 2006;110(33):16185–16188. doi: 10.1021/jp064054s 16913738

[220] Fujigaya T, Okamoto M, Nakashima N. Design of an assembly of pyridine-containing polybenzimidazole, carbon nanotubes and Pt nanoparticles for a fuel cell electrocatalyst with a high electrochemically active surface area. Carbon. 2009;47(14):3227–3232. doi: 10.1016/j.carbon.2009.07.038

[221] Berber MR, Hafez IH, Fujigaya T, et al. A highly durable fuel cell electrocatalyst based on double-polymer-coated carbon nanotubes. Sci Rep. 2015;5(1):16711. doi: 10.1038/srep16711 26594045 PMC4655398

[222] Berber MR, Fujigaya T, Sasaki K, et al. Remarkably durable high temperature polymer electrolyte fuel cell based on poly(vinylphosphonic acid)-doped polybenzimidazole [Article]. Sci Rep. 2013;3(1):1764. doi: 10.1038/srep01764

[223] Kim C, Fujigaya T, Nakashima N. One-pot synthesis of gold-platinum core-shell nanoparticles on polybenzimidazole-decorated carbon nanotubes [Article]. Chem Lett. 2014;43(11):1737–1739. doi: 10.1246/cl.140663

[224] Verma S, Hamasaki Y, Kim C, et al. Insights into the low overpotential electroreduction of CO2 to CO on a supported gold catalyst in an alkaline flow electrolyzer. ACS Energy Lett. 2018;3(1):193–198. doi: 10.1021/acsenergylett.7b01096

[225] Fujigaya T, Kim C, Matsumoto K, et al. Palladium-based anion-exchange membrane fuel cell using KOH-doped polybenzimidazole as the electrolyte. ChemPluschem. 2014;79(3):400–405. doi: 10.1002/cplu.201300377 31986600

[226] Fujigaya T, Shi Y, Yang J, et al. A highly efficient and durable carbon nanotube-based anode electrocatalyst for water electrolyzers. J Mater Chem A. 2017;5(21):10584–10590. doi: 10.1039/C7TA01318C

[227] Hafez IH, Berber MR, Fujigaya T, et al. High electronic conductivity and air stability of ultrasmall copper-metal nanoparticles supported on pyridine-based polybenzimidazole carbon nanotube composite [Article]. ChemCatchem. 2017;9(22):4282–4286. doi: 10.1002/cctc.201700921

[228] Yang J, Fujigaya T, Nakashima N. Decorating unoxidized-carbon nanotubes with homogeneous Ni-Co spinel nanocrystals show superior performance for oxygen evolution/reduction reactions. Sci Rep. 2017;7(1):45384. doi: 10.1038/srep45384 28358114 PMC5371823

[229] Jhong HRM, Tornow CE, Kim C, et al. Gold nanoparticles on polymer-wrapped carbon nanotubes: an efficient and selective catalyst for the electroreduction of CO2 [Article]. Chemphyschem. 2017;18(22):3274–3279. doi: 10.1002/cphc.201700815 28985010

[230] Sari SR, Tsushida M, Sato T, et al. Highly sensitive detection of phosphate using well-ordered crystalline cobalt oxide nanoparticles supported by multi-walled carbon nanotubes [Article]. Mater Adv. 2022;3(4):2018–2025. doi: 10.1039/D1MA01097B

[231] Du H-Y, Wang C-H, Yang C-S, et al. A high performance polybenzimidazole–CNT hybrid electrode for high-temperature proton exchange membrane fuel cells. J Mater Chem A. 2014;2(19):7015–7019. doi: 10.1039/C4TA00091A

[232] Berber MR, Alenad AM, Althubiti NA, et al. Bipyridine-based polybenzimidazole as a nitrogen-rich ionomer and a platinum nanoparticle support for enhanced fuel cell performance. Fuel. 2022;312:122954. doi: 10.1016/j.fuel.2021.122954

[233] Yu X, Luo F, Yang Z. Bottom-up design of a stable CO-tolerant platinum electrocatalyst with enhanced fuel cell performance in direct methanol fuel cells. RSC Adv. 2016;6(101):98861–98866. doi: 10.1039/C6RA24025A

[234] Yang Z, Li J, Ling Y, et al. Bottom-up design of high-performance Pt electrocatalysts supported on carbon nanotubes with homogeneous ionomer distribution. ChemCatchem. 2017;9(17):3307–3313. doi: 10.1002/cctc.201700587

[235] Kato M, Nakahoshiba R, Ogura K, et al. Electronic effects of nitrogen atoms of supports on Pt–Ni rhombic dodecahedral nanoframes for oxygen reduction. ACS Appl Energy Mater. 2020;3(7):6768–6774. doi: 10.1021/acsaem.0c00903

[236] Li Z-F, Xin L, Yang F, et al. Hierarchical polybenzimidazole-grafted graphene hybrids as supports for Pt nanoparticle catalysts with excellent PEMFC performance. Nano Energy. 2015;16:281–292. doi: 10.1016/j.nanoen.2015.06.031

[237] Gebremariam TT, Chen F, Kou B, et al. PdAgRu nanoparticles on polybenzimidazole wrapped CNTs for electrocatalytic formate oxidation. Electrochim Acta. 2020;354:136678. doi: 10.1016/j.electacta.2020.136678

[238] Zhang Y, Wang Y, Yu J, et al. Polybenzimidazole assisted fabrication of multiwalled carbon nanotube buckypapers and their silver nanoparticle hybrids. RSC Adv. 2014;4(68):35904–35913. doi: 10.1039/C4RA06445C

[239] Du H-Y, Yang C-S, Hsu H-C, et al. Pulsed electrochemical deposition of Pt NPs on polybenzimidazole-CNT hybrid electrode for high-temperature proton exchange membrane fuel cells. Int J Hydrogen Energy. 2015;40(41):14398–14404. doi: 10.1016/j.ijhydene.2015.04.131

[240] Yang J, Cheng J, Tao J, et al. Wrapping multiwalled carbon nanotubes with anatase titanium oxide for the electrosynthesis of glycolic acid. ACS Appl Nano Mater. 2019;2(10):6360–6367. doi: 10.1021/acsanm.9b01357

[241] Fujigaya T, Kim C, Matsumoto K, et al. Effective anchoring of Pt-nanoparticles onto sulfonated polyelectrolyte-wrapped carbon nanotubes for use as a fuel cell electrocatalyst. Polym J. 2013;45(3):326–330. doi: 10.1038/pj.2012.145

[242] Yang Z, Nakashima N. An electrocatalyst based on carbon nanotubes coated with poly(vinylpyrrolidone) shows a high tolerance to carbon monoxide in a direct methanol fuel cell. ChemCatchem. 2016;8(3):600–606. doi: 10.1002/cctc.201501060

[243] Yoo J, Lee S, Lee CK, et al. Homogeneous decoration of zeolitic imidazolate framework-8 (zif-8) with core–shell structures on carbon nanotubes. RSC Adv. 2014;4(91):49614–49619. doi: 10.1039/C4RA06792D

[244] Lu Q, Fang J, Yang J, et al. A polyimide ion-conductive protection layer to suppress side reactions on Li4Ti5O12 electrodes at elevated temperature. RSC Adv. 2014;4(20):10280–10283.

[245] Wu D, Jayawickrama SM, Fujigaya T. Effect of polymer-coating on acetylene Black for durability of polymer electrolyte membrane fuel cell. J Power Sources. 2022;549:232079. doi: 10.1016/j.jpowsour.2022.232079

[246] Fujigaya T, Saito C, Han Z, et al. Ionomer grafting to polymer-wrapped carbon nanotubes for polymer electrolyte membrane fuel cell electrocatalyst. Chem Lett. 2017;46(11):1660–1663. doi: 10.1246/cl.170744

[247] Fujigaya T, Uchinoumi T, Kaneko K, et al. Design and synthesis of nitrogen-containing calcined polymer/carbon nanotube hybrids that act as a platinum-free oxygen reduction fuel cell catalyst. Chem Commun. 2011;47(24):6843–6845. doi: 10.1039/c1cc11303h 21562659

[248] Fujigaya T, Morita J, Nakashima N. Grooves of bundled single‐walled carbon nanotubes dramatically enhance the activity of the oxygen reduction reaction. ChemCatchem. 2014;6(11):3169–3173. doi: 10.1002/cctc.201402565

[249] Balan BK, Manissery AP, Chaudhari HD, et al. Polybenzimidazole mediated n-doping along the inner and outer surfaces of a carbon nanofiber and its oxygen reduction properties. J Mater Chem. 2012;22(44):23668–23679. doi: 10.1039/c2jm35033e

[250] Fujigaya T, Kanamori R, Hirata S, et al. Effect of nitrogen-containing polymer wrapped around carbon nanotubes for Li–O2 battery cathode. Polym J. 2019;51(9):921–927. doi: 10.1038/s41428-019-0207-2

[251] Lu Y, Wen Z, Jin J, et al. Mesoporous carbon nitride loaded with Pt nanoparticles as a bifunctional air electrode for rechargeable lithium-air battery. J Solid State Electrochem. 2012;16(5):1863–1868. doi: 10.1007/s10008-012-1640-8

[252] Bose S, Kuila T, Mishra AK, et al. Carbon-based nanostructured materials and their composites as supercapacitor electrodes. J Mater Chem. 2012;22(3):767–784. doi: 10.1039/C1JM14468E

[253] Nyholm L, Nyström G, Mihranyan A, et al. Toward flexible polymer and paper-based energy storage devices. Adv Mater. 2011;23(33):3751–3769. doi: 10.1002/adma.201004134 21739488

[254] Zeng J, Yang F, Yang S, et al. Anchoring polyaniline molecule on 3d carbon nanotube meshwork as self-standing cathodes for advanced rechargeable zinc ion batteries. J Power Sources. 2021;508:230329. doi: 10.1016/j.jpowsour.2021.230329

[255] Liu P, Yan J, Guang Z, et al. Recent advancements of polyaniline-based nanocomposites for supercapacitors. J Power Sources. 2019;424:108–130. doi: 10.1016/j.jpowsour.2019.03.094

[256] Chen C, Wei S, Zhang Q, et al. High-performance VO2/CNT@PANI with core–shell construction enable printable in-planar symmetric supercapacitors. J Colloid Interface Sci. 2024;664:53–62. doi: 10.1016/j.jcis.2024.03.012 38458055

[257] Yu C, An J, Zhou R, et al. Microstructure design of carbonaceous fibers: a promising strategy toward high-performance weaveable/wearable supercapacitors. Small. 2020;16(25):2000653. doi: 10.1002/smll.202000653 32432831

[258] Shoaib Tahir M, Kainat I, Ghazanfar H, et al. Flexible electrodes for high-performance energy storage: materials, conductivity optimization, and scalable fabrication. Nanoscale. 2025;17(31):18016–18048. doi: 10.1039/D5NR01647A 40699900

[259] Jiang Y, Ou J, Luo Z, et al. High capacitive antimonene/CNT/PANI free-standing electrodes for flexible supercapacitor engaged with self-healing function. Small. 2022;18(25):2201377. doi: 10.1002/smll.202201377 35603958

[260] Yomogida Y, Tanaka T, Zhang M, et al. Industrial-scale separation of high-purity single-chirality single-wall carbon nanotubes for biological imaging. Nat Commun. 2016;7(1):12056. doi: 10.1038/ncomms12056 27350127 PMC4931232

[261] Iizumi Y, Yudasaka M, Kim J, et al. Oxygen-doped carbon nanotubes for near-infrared fluorescent labels and imaging probes. Sci Rep. 2018;8(1):6272. doi: 10.1038/s41598-018-24399-8 29674647 PMC5908862

[262] Yudasaka M, Yomogida Y, Zhang M, et al. Fasting-dependent vascular permeability enhancement in brown adipose tissues evidenced by using carbon nanotubes as fluorescent probes. Sci Rep. 2018;8(1):14446. doi: 10.1038/s41598-018-32758-8 30262832 PMC6160465

[263] Hirata E, Yudasaka M, Ushijima N, et al. Fate of carbon nanotubes locally implanted in mice evaluated by near-infrared fluorescence imaging: implications for tissue regeneration. ACS Appl Nano Mater. 2019;2(3):1382–1390. doi: 10.1021/acsanm.8b02267

[264] Liu Z, Davis C, Cai W, et al. Circulation and long-term fate of functionalized, biocompatible single-walled carbon nanotubes in mice probed by Raman spectroscopy. Proc Natl Acad Sci USA. 2008;105(5):1410–1415. doi: 10.1073/pnas.0707654105 18230737 PMC2234157

[265] Prencipe G, Tabakman SM, Welsher K, et al. Peg branched polymer for functionalization of nanomaterials with ultralong blood circulation. J Am Chem Soc. 2009;131(13):4783–4787. doi: 10.1021/ja809086q 19173646 PMC2827329

[266] Robinson JT, Hong G, Liang Y, et al. In vivo fluorescence imaging in the second near-infrared window with long circulating carbon nanotubes capable of ultrahigh tumor uptake. J Am Chem Soc. 2012;134(25):10664–10669. doi: 10.1021/ja303737a 22667448 PMC3471786

[267] Yudasaka M, Yomogida Y, Zhang M, et al. Near-infrared photoluminescent carbon nanotubes for imaging of brown fat. Sci Rep. 2017;7(1):44760. doi: 10.1038/srep44760 28317858 PMC5357894

[268] Tsutsumi Y, Fujigaya T, Nakashima N. Polymer synthesis inside a nanospace of a surfactant-micelle on carbon nanotubes: creation of highly-stable individual nanotubes/ultrathin cross-linked polymer hybrids [Article]. RSC Adv. 2014;4(12):6318–6323.

[269] Tsutsumi Y, Fujigaya T, Nakashima N. Requirement for the formation of crosslinked polymers on single-walled carbon nanotubes using vinyl monomers [Article]. Chem Lett. 2016;45(3):274–276. doi: 10.1246/cl.151086

[270] Mori K, Kawaguchi M, Fujigaya T, et al. Polymer-coated carbon nanotubes as a molecular heater platform for hyperthermic therapy [Article]. J Hard Tissue Biol. 2018;27(2):139–146. doi: 10.2485/jhtb.27.139

[271] Nagai Y, Tsutsumi Y, Nakashima N, et al. Synthesis of single-walled carbon nanotubes coated with thiol-reactive gel via emulsion polymerization [Article]. J Am Chem Soc. 2018;140(27):8544–8550. doi: 10.1021/jacs.8b03873 29906397

[272] Nagai Y, Nakamura K, Ohno J, et al. Antibody-conjugated gel-coated single-walled carbon nanotubes as photothermal agents [Article]. ACS Appl Bio Mater. 2021;4(6):5049–5056. doi: 10.1021/acsabm.1c00299 35007053

[273] Law SSY, Liou G, Nagai Y, et al. Polymer-coated carbon nanotube hybrids with functional peptides for gene delivery into plant mitochondria [Article]. Nat Commun. 2022;13(1). doi: 10.1038/s41467-022-30185-y PMC9110379 35577779

[274] Nagai Y, Nakamura K, Yudasaka M, et al. Radical polymer grafting on the surface of single-walled carbon nanotubes enhances photoluminescence in the near-infrared region: implications for bioimaging and biosensing [Article]. ACS Appl Nano Mater. 2020;3(9):8840–8847. doi: 10.1021/acsanm.0c01561

[275] Nagai Y, Hamano R, Nakamura K, et al. Bright NIR-II fluorescence from biocompatible gel-coated carbon nanotubes for in vivo imaging [Article]. Carbon. 2024;218:118728. doi: 10.1016/j.carbon.2023.118728

[276] Hamano R, Tanaka N, Fujigaya T. Size exclusion chromatography-based length sorting of single-walled carbon nanotubes stably coated with cross-linked polymers [Article]. Mater Adv. 2024;5(6):2482–2490. doi: 10.1039/D3MA01069D

[277] Khripin CY, Tu X, Heddleston JM, et al. High-resolution length fractionation of surfactant-dispersed carbon nanotubes. Anal Chem. 2013;85(3):1382–1388. doi: 10.1021/ac303349q 23259532

[278] Zaumseil J. Luminescent defects in single-walled carbon nanotubes for applications. Adv Optical Mater. 2022;10(2):2101576. doi: 10.1002/adom.202101576

